# PP2Ac/STRN4 negatively regulates STING-type I IFN signaling in tumor-associated macrophages

**DOI:** 10.1172/JCI162139

**Published:** 2023-03-15

**Authors:** Winson S. Ho, Isha Mondal, Beisi Xu, Oishika Das, Raymond Sun, Pochin Chiou, Xiaomin Cai, Foozhan Tahmasebinia, Elizabeth McFadden, Caren Yu-Ju Wu, Zhihao Wu, William Matsui, Michael Lim, Zhipeng Meng, Rongze Olivia Lu

**Affiliations:** 1 Department of Neurosurgery, Dell Medical School, The University of Texas at Austin, Austin, Texas, USA.; 2 Center for Applied Bioinformatics, St. Jude Children’s Research Hospital, Memphis, Tennessee, USA.; 3 Department of Molecular and Cellular Pharmacology, University of Miami Miller School of Medicine, Miami, Florida, USA.; 4 Department of Biological Sciences, Southern Methodist University, Dallas, Texas, USA.; 5 Department of Neurosurgery, Stanford University, Stanford, California, USA.; 6 Department of Oncology, Dell Medical School, The University of Texas at Austin, Austin, Texas, USA.; 7 Department of Molecular Sciences, University of Texas at Austin, Austin, Texas, USA.; 8 Helen Diller Comprehensive Cancer Center, UCSF, San Francisco, California, USA.

**Keywords:** Immunology, Oncology, Cancer immunotherapy, Macrophages

## Abstract

Stimulator of IFN genes type I (STING-Type I) IFN signaling in myeloid cells plays a critical role in effective antitumor immune responses, but STING agonists as monotherapy have shown limited efficacy in clinical trials. The mechanisms that downregulate STING signaling are not fully understood. Here, we report that protein phosphatase 2A (PP2A), with its specific B regulatory subunit Striatin 4 (STRN4), negatively regulated STING-Type I IFN in macrophages. Mice with macrophage PP2A deficiency exhibited reduced tumor progression. The tumor microenvironment showed decreased immunosuppressive and increased IFN-activated macrophages and CD8^+^ T cells. Mechanistically, we demonstrated that Hippo kinase MST1/2 was required for STING activation. STING agonists induced dissociation of PP2A from MST1/2 in normal macrophages, but not in tumor conditioned macrophages. Furthermore, our data showed that STRN4 mediated PP2A binding to and dephosphorylation of Hippo kinase MST1/2, resulting in stabilization of YAP/TAZ to antagonize STING activation. In human patients with glioblastoma (GBM), YAP/TAZ was highly expressed in tumor-associated macrophages but not in nontumor macrophages. We also demonstrated that PP2A/STRN4 deficiency in macrophages reduced YAP/TAZ expression and sensitized tumor-conditioned macrophages to STING stimulation. In summary, we demonstrated that PP2A/STRN4-YAP/TAZ has, in our opinion, been an unappreciated mechanism that mediates immunosuppression in tumor-associated macrophages, and targeting the PP2A/STRN4-YAP/TAZ axis can sensitize tumors to immunotherapy.

## Graphical abstract

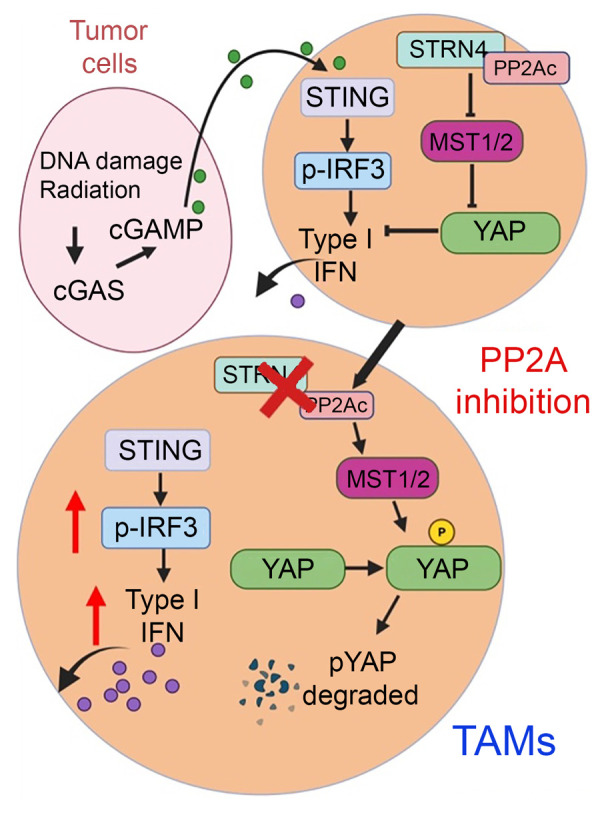

## Introduction

Cyclic GMP–AMP synthase/stimulator of IFN genes (cGAS/STING) is a critical sensor for cytosolic double stranded DNA (dsDNA) to elicit antitumor immunity (1–4). cGAS binding to dsDNA leads to the formation of 2′,3′-cyclic GMP–AMP (cGAMP) that activates STING and induces the phosphorylation of IFN regulatory factor 3 (IRF3) to promote Type I IFN production and antitumor immune responses (3–5). Both tumor cells and myeloid cells express cGAS/STING, but accumulating evidence suggests that cGAMP is primarily produced by tumor cells and released as an immunotransmitter to activate STING in myeloid cells and stimulate antitumor immunity by triggering Type I IFN production (6–8). Despite promising preclinical studies, several small-molecule agonists of STING have shown limited clinical efficacy in early phase clinical trials (9). It is possible that these agonists cannot fully activate and sustain STING-Type I IFN signaling because the mechanisms that normally attenuate STING signaling remain engaged in the tumor microenvironment. The steps involved in STING activation are well characterized, but the mechanisms that serve to downregulate STING are not well understood. As specific phosphorylation events are required for STING activation, it is likely that dephosphorylation is involved in attenuating signaling.

Protein phosphatase 2A (PP2A) is a major protein phosphatase that accounts for 50%–70% of the total serine/threonine phosphatase activity in eukaryotic cells to counterbalance the regulatory effects of kinases in modulating numerous signaling pathways (10–12). PP2A consists of various subunits, such as regulatory, B; scaffolding, A; and catalytic,C subunits. Different combinations of A, B and C subunits can lead to 60 different PP2A holoenzymes with distinct functions in different cell types (11, 12). The specificity of PP2A holoenzymes is determined by a heterogenous family of regulatory B subunits. Our group was the first to report that pharmacological inhibition of the PP2A catalytic subunit C (PP2Ac) enhances the efficacy of immune checkpoint blockade in multiple PD-1-resistant mouse tumor models (13, 14). However, given the ubiquity of PP2A expression in many cell types and the promiscuity of PP2A involvement in many cellular pathways, the mechanisms of how PP2A regulates antitumor immunity are unclear. Tumor-associated macrophages (TAMs) are the predominant myeloid cells in the tumor environment and are associated with poor prognosis in cancer. TAMs can promote immunosuppression and inhibit antitumor T cell responses, thereby limiting the efficacy of checkpoint inhibitors (15–18). Previous studies have demonstrated that PP2A plays a critical role in regulating TLR-mediated Type-I IFN and NFkB signaling in macrophage response to viral infections (19, 20). However, the role of PP2A in TAMs, and, in particular, STING-mediated Type I IFN signaling is unexplored.

In this study, we present biochemical, genetic, and functional evidence that PP2A with its specific regulatory B subunit, STRN4, negatively regulates STING–Type I IFN signaling in macrophages. Mice with PP2Ac deficiency in macrophages exhibited reduced tumor growth, increased numbers of tumor-infiltrating CD8^+^ T cells, and reduced numbers of immunosuppressive macrophages. Macrophage PP2Ac deficiency also synergizes with STING agonists, radiation, and checkpoint blockade in multiple syngeneic tumor models. Single-cell RNA-Seq (scRNA-Seq) demonstrated that macrophage-specific loss of PP2Ac resulted in complex remodeling of the immune landscape with enhanced Type I IFN signature in TAMs and an increased adaptive immune response. STRN4 has been implicated in biochemical studies to regulate the Hippo-Yes-associated protein (Hippo-YAP) pathway (21), which has an established role in tumorigenesis. However, the function of STRN4 has not been described in immune cells and the role of Hippo-YAP pathways has not been explored in TAMs. We found that the Hippo kinase mammalian STE20-like protein kinase (MST1/2), a negative regulator of YAP and transcriptional coactivator with PDZ-binding motif (TAZ), is required for STING activation. Mechanistically, STRN4 associates with PP2Ac to dephosphorylate Hippo kinase MST1/2, resulting in stabilization of YAP/TAZ to antagonize STING activation. We also found that tumor significantly upregulated YAP/TAZ expression in TAMs, resulting in suppression of Type I IFN signaling in the context of cGAS-STING stimulation. Thus, PP2A/STRN4-Hippo-YAP/TAZ signaling is critical in regulating STING-Type I IFN in TAMs. Our work provides the rationale for targeting this pathway to enhance antitumor immunity by combination with other STING-activating strategies.

## Results

### PP2Ac negatively regulates STING-Type I IFN signaling pathway.

**Figure 1:**
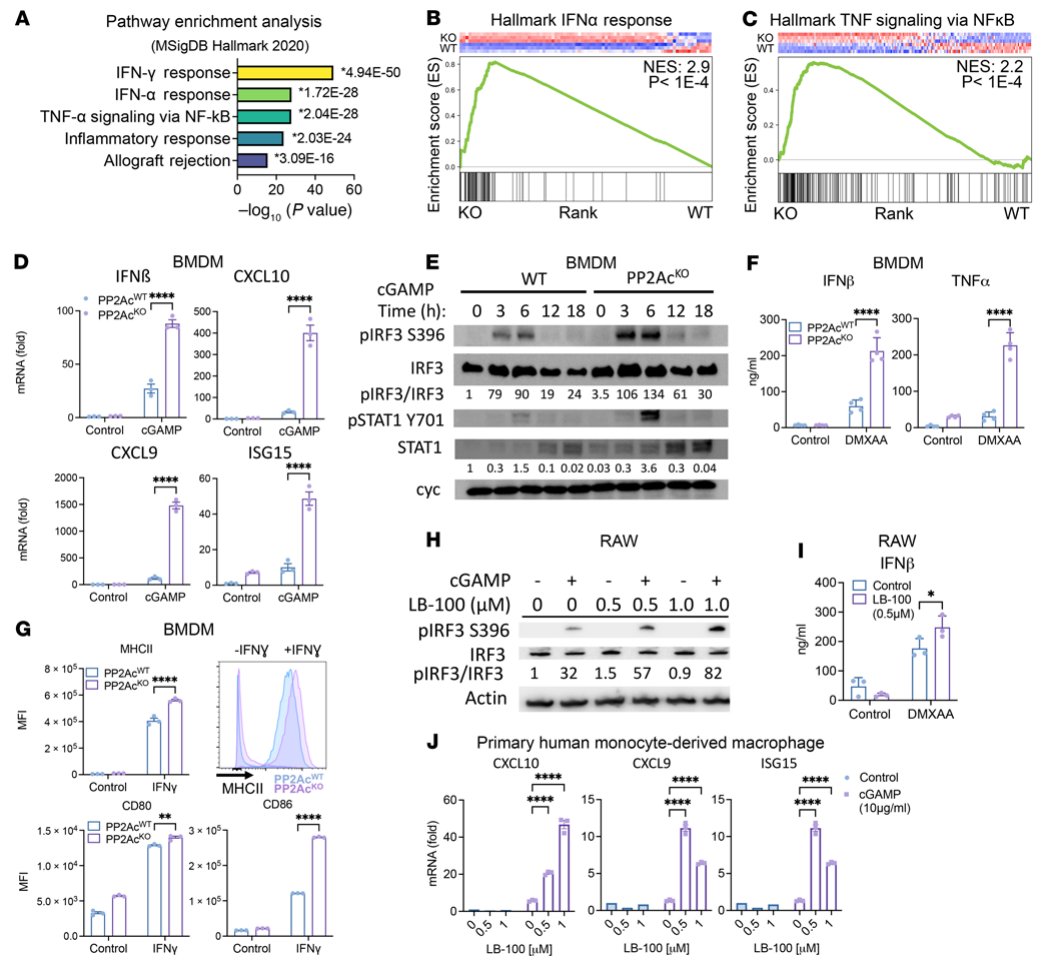
PP2A negatively regulates STING-Type I IFN signaling pathway. (**A**) Pathway enrichment analysis of RNA-Seq of PP2Ac^KO^ and PP2Ac^WT^ BMDM treated with cGAMP (10 μg/mL) for 4 hours (*n* = 3 per group) showing the top 5 enriched pathways ranked with highest –log10 *P* value using differentially upregulated genes in PP2Ac^KO^ compared with PP2Ac^WT^ BMDM (Log_2_ fold change (log_2_FC) > 1, FDR < 0.01). * indicates the *P* value for each individual pathway. (**B** and **C**) GSEA plots for Type I IFN (**B**) and TNF (**C**) signatures between cGAMP-treated PP2Ac^KO^ versus PP2Ac^WT^ BMDM. (**D**) BMDM were harvested 4 hours after cGAMP stimulation (10 μg/mL). Expression of IFNβ and IFN response genes (CXCL10, CXCL9, and ISG15) were measured via reverse transcription PCR. (**E**) Protein expression of BMDM was analyzed by immunoblotting after cGAMP (10 μg/mL) treatment. (**F**) PP2Ac^KO^ and PP2Ac^WT^ BMDM were stimulated with DMXAA (10 μg/mL) for 48 hours, cytokine concentrations were measured in culture supernatant. (**G**) PP2Ac^KO^ and PP2Ac^WT^ BMDM were treated with IFNγ (10 ng/mL) for 24 hours, expressions of CD80, CD86, and MHCII were measured by FACS. Representative FACS plot of MHCII expression **±** IFNγ treatment. (**H**) RAW cells were pretreated with the PP2A inhibitor LB-100 for 2 hours before stimulated with cGAMP (10 μg/mL) for 4 hours. Protein expression was analyzed by immunoblotting. (**I**) RAW cells were pretreated with LB-100 for 2 hours before stimulated with DMXAA (10 μg/mL) for 48 hours. Cytokine concentrations were measured in culture supernatant. (**J**) PBMCs were treated with M-CSF (50 ng/mL) for 6 days to derive macrophages. Cells were then pretreated with LB-100 at the indicated dosage for 1.5 hours prior to cGAMP (10 μg/mL) treatment. Expression of IFN response genes (CXCL10, CXCL9, and ISG15) were measured via real time PCR. Data are from 1 experiment representative of at least 2 (**B**–**I**) and 1 (**J**) independent experiments with similar results. Error bars depict SEM. *P* values were calculated by unpaired 2-tailed *t* test *P < 0.05,**P < 0.01, ***P < 0.001, ****P < 0.0001.

To address the effect of PP2Ac on STING signaling in macrophages, we chose the *LysM^cre^PP2Ac^fl/fl^* mice. These mice carry floxP sites that flank exon 1 of *ppp2ca,* have *cre* expression under the Lysozyme 2 (*LysM*) promoter, and have a myeloid lineage, specifically the macrophages, that have a deficiency in PP2Ac (22). We generated bone marrow-derived macrophages (BMDM) from *LysM^cre^PP2Ac^fl/fl^* and WT mice and treated them with the STING agonist cGAMP. RNA-Seq was then performed to identify global gene expression–profile changes. Pathway enrichment analysis demonstrated that IFN and TNF signaling pathways were among the highest enriched differentially expressed gene sets between PP2Ac^KO^ and PP2Ac^WT^ BMDM in response to cGAMP (Figure 1A). Since both IFN and TNF are known downstream signaling of STING activation, this result is consistent with PP2Ac-deficiency mediated STING activation. Gene set enrichment analysis (GSEA) confirmed the upregulation of gene signatures associated with Type I IFN (Figure 1B) and TNF (Figure 1C) responses in PP2Ac^KO^ compared with PP2Ac^WT^ BMDM following cGAMP stimulation. The implication of Type II (γ) IFN signaling in pathway enrichment analysis is likely due to overlapping Type I and Type II IFN gene sets and not due to IFN-γ receptor (IFNGR) activation. Indeed, BMDMs were stimulated with cGAMP in isolation without other in vitro sources of Type II IFN, which is primarily secreted by lymphocytes rather than macrophages. Therefore, the implication of Type II (γ) IFN signaling in pathway enrichment analysis is likely due to overlapping Type I and Type II IFN gene sets and not due to IFN-γ–receptor (IFNGR) activation. Next, we confirmed using RT-PCR that the expression of IFNβ and 3 critical IFN-stimulated genes (CXCL10, CXCL9, and ISG15) (23) were upregulated in cGAMP-stimulated PP2Ac^KO^ BMDM (Figure 1D). Furthermore, we examined the time course of p-IRF3 and p-STAT1 protein expression following cGAMP treatment. p-IRF3 is the downstream mediator of STING activation leading to transcription of IFNα/β, and p-STAT1 is activated by the IFNα/β receptor IFNAR, by autocrine Type I IFN stimulation. Compared with PP2Ac^WT^, PP2Ac^KO^ BMDM had amplified activation of both pIRF3 and pSTAT1 following cGAMP stimulation. Response peaked at 6 hours, but the signal remained elevated in PP2Ac^KO^ compared with PP2Ac^WT^ at 18 hours after stimulation (Figure 1E). Production of IFNβ and TNF cytokines in the culture supernatant in response to STING stimulation was enhanced in PP2Ac^KO^ compared with PP2Ac^WT^ BMDM (Figure 1F). We also asked if PP2Ac^KO^ in BMDM enhanced antigen presenting phenotype in classically activated M1 condition by measuring the expression of MHCII, CD80, and CD86 (Figure 1G and Supplemental Figure 1A; supplemental material available online with this article; https://doi.org/10.1172/JCI162139DS1), which are essential for macrophage activation and antigen presentation. PP2Ac^KO^ BMDM treated with STING agonists had increased CD86 expression (Supplemental Figure 1B) compared with the control. We also tested the effect of pharmacologic PP2Ac inhibition using a small molecule inhibitor, LB-100. p-IRF3 (Figure 1H) and IFNβ cytokine production (Figure 1I) in mouse macrophage RAW cells were enhanced with STING agonist stimulation. To generalize our findings in human macrophages, we also generated PP2Ac^KO^ human ThP-1 cell lines using CRISPR/Cas9 gene-KO technique and confirmed that PP2Ac protein expression was absent in these cells (Supplemental Figure 2A). THP-1 cells were differentiated into macrophages by phorbol myristate acetate (PMA) for 24 hours before stimulated with STING agonists for 4 hours. Expression of IFNβ and IFN-stimulated genes were upregulated in PP2Ac^KO^ THP-1 differentiated macrophages compared with control cells (Supplemental Figure 3A). Protein expression of pIRF3 was also enhanced in PP2Ac^KO^ compared with control cells following cGAMP treatment (Supplemental Figure 3B). We further tested the effect of LB-100 on primary human macrophages. Human PBMC–derived monocytes were treated with M-CSF for 6 days to induce macrophage differentiation. Cells were then treated with LB-100 for 2 hours prior to stimulation with cGAMP. After 4 hours, expressions of IFN-stimulated genes CXCL10, CXCL9, and ISG15 were found to be significantly enhanced in LB-100–treated macrophages (Figure 1J). Cumulatively, these results demonstrated that genetic or pharmacological inhibition of PP2Ac consistently enhanced STING-Type I IFN signaling in human and murine macrophage cells.

### Deficiency of PP2Ac in macrophages reduces tumor growth and alters tumor immune microenvironment.

**Figure 2:**
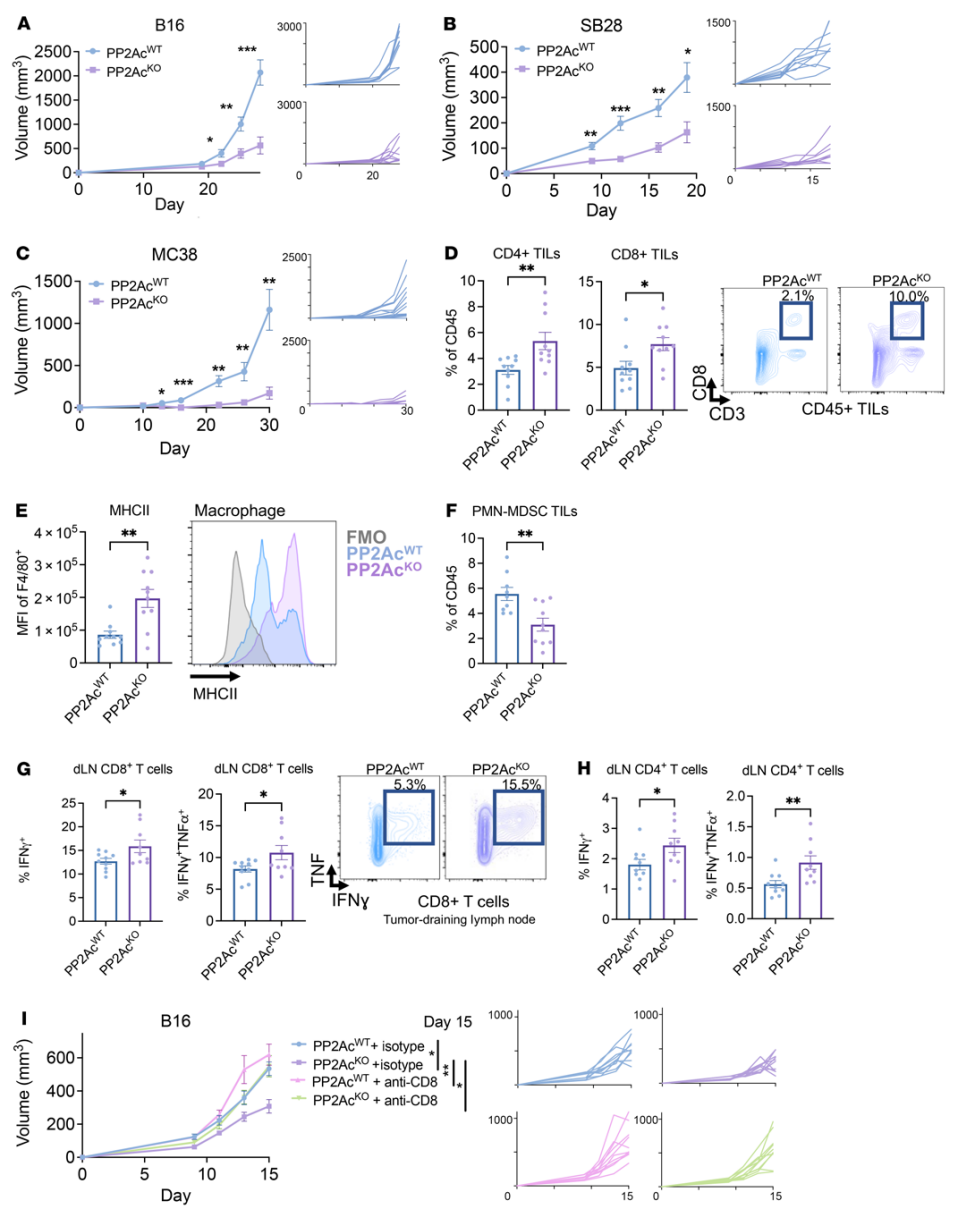
Macrophage PP2Ac deficiency reduces tumor growth and alters the tumor immune microenvironment. (**A**–**C**) *LysM^cre^PP2Ac^fl/fl^* or WT *C57BL/6* mice were inoculated with 0.1 × 10^6^ (**A**) B16, (**B**) SB28, or (**C**) 1 × 10^6^ MC38 cells s.c. (*n* = 8–10). (**D**–**G**) B16 tumors were implanted s.c. in *LysM^cre^PP2Ac^fl/fl^* or WT mice. Mice were euthanized on day 10. Tumors were harvested for tumor infiltrating leukocyte (TIL) profiling (**D**–**F**) and tumor-draining lymph node (tumor-dLN) (**G** and **H**) by flow cytometry (*n* = 9–10). (**D**) Quantification of CD4^+^ and CD8^+^ TILs and representative FACS plot. (**E**) Quantification of MHCII^+^ expression in tumor infiltrating macrophages (F4/80^+^) with representative FACS plot. (**F**) Quantification of Ly6G^+^Ly6C^lo^ PMN-MDSC in TILs. (**G**–**H**) Quantitation of IFNγ-producing or IFNγ/TNF dual–producing dLN-resident CD8^+^ (**G**) and CD4^+^ (**H**) T cells as percentages of total CD8^+^ and CD4^+^ T cells, respectively. IFNγ and/or TNF production was stimulated exvivo with PMA/ionomycin in conjunction with protein transport inhibitor for 4 hours prior to staining. Representative FACS plots of dLN CD8^+^ T cells after stimulation. (**I**) *LysM^cre^PP2Ac^fl/fl^* or WT *C57BL/6* mice were treated with anti-CD8 depletion antibody or isotype control. Mice were given 250 μg i.p. on day –3, –2, and –1, then inoculated with 0.1 × 10^6^ B16 cells s.c. (day 0) (*n* = 8). Depleting antibody or isotype was then given 2 × per week until endpoint. Data are from 1 experiment representative of at least 2 (**A**–**H**) and 1 (**I**) independent experiments with similar results. Error bars depict SEM. *P* values were calculated by unpaired 2-tailed *t* test (*P < 0.05, **P < 0.01, ***P < 0.001, ****P < 0.0001).

To test our hypothesis that PP2Ac^KO^ in macrophages can enhance antitumor immunity, we implanted B16 melanoma, SB28 glioma, and MC38 colon tumor cells s.c. in *LysM^cre^PP2Ac^fl/fl^* and WT mice. We chose these cell lines because of their variable range of intrinsic immunogenicity. Tumor growth was all significantly reduced in LysM^cre^PP2Ac^fl/fl^ mice (Figure 2, A–C), suggesting that macrophage-specific PP2Ac deficiency can induce a potent antitumor effect. We then assessed the functional consequence of macrophage PP2Ac deletion on tumor-infiltrating leukocytes (TILs) and tumor-draining lymph node-resident (tumor-dLN-resident) T cells in B16 melanoma. Ten days after implantation, tumors and tumor-dLN were harvested and analyzed by flow cytometry. We found increased infiltration of CD8^+^ and CD4^+^ T cells in the tumor (Figure 2D and Supplemental Figure 4A) and systemically in the spleen (Supplemental Figure 4B). We did not find a significant change in total F4/80 macrophage tumor infiltration (Supplemental Figure 4C). However, consistent with our in vitro findings, we found significantly enhanced expression of MHCII in F4/80^+^ macrophages (Figure 2E), suggesting an enhanced proinflammatory phenotype in tumor-infiltrating macrophages. In addition, the frequency of immunosuppressive polymorphonuclear myeloid-derived suppressor cells (PMN-MDSC) was significantly decreased (Figure 2F). In tumor-dLN, there was an increased frequency of resident CD4^+^ T cells (Supplemental Figure 4E) and enhanced IFNγ and IFNγ^+^ TNF producing CD8^+^ and CD4^+^ T cells in mice with macrophage-specific PP2Ac^KO^ (Figure 2, G and H, and Supplemental Figure 4F). To confirm the generalizability of the immunomodulatory effect of macrophage-specific PP2A^KO^ on TILs in other cancer models, we also examined orthotopic intracranial (i.c.) GL261 glioma. The brain tumor microenvironment is known to be more immunosuppressive compared with s.c. tumors (24). We similarly found increased CD8^+^ and CD4^+^ T cell infiltration (Supplemental Figure 5, A and B). The frequency of monocytic derived macrophages (CD11b^+^CD49d^+^) — a subpopulation of TAMs in brain tumors known to correlate with the clinical outcome (18) — was significantly decreased in *LysM^cre^PP2Ac^fl/fl^* mice (Supplemental Figure 5C). TAMs were found to have increased expression of activation markers MHCII and CD86 and decreased immunosuppressive marker CD206 (25, 26) in *LysM^cre^PP2Ac^fl/fl^* mice (Supplemental Figure 5D). The fact that we observed increased tumor infiltration of cytotoxic T cells suggested that macrophage PP2Ac deficiency can elicit enhanced adaptive antitumor immunity. We asked whether the increased CD8^+^ T cell response was required for the antitumor effect in *LysM^cre^PP2Ac^fl/fl^* mice. We performed systemic CD8^+^ T cell depletion by treating WT and *LysM^cre^PP2Ac^fl/fl^* mice with isotype control or anti-CD8 depleting antibody prior to tumor implantation and throughout the study. CD8 depletion completely abolished the benefit of *LysM^cre^PP2Ac^fl/fl^* mice, suggesting that CD8-mediated adaptive immune response was required for the beneficial effects of macrophage PP2Ac KO (Figure 2I). In summary, these data suggest that macrophage PP2Ac–deficiency enhanced T cell effector function by remodeling the myeloid compartment of the tumor immune microenvironment.

### Deficiency of PP2Ac in macrophages sensitizes tumors to STING agonists, radiation, and immune checkpoint blockade.

**Figure 3:**
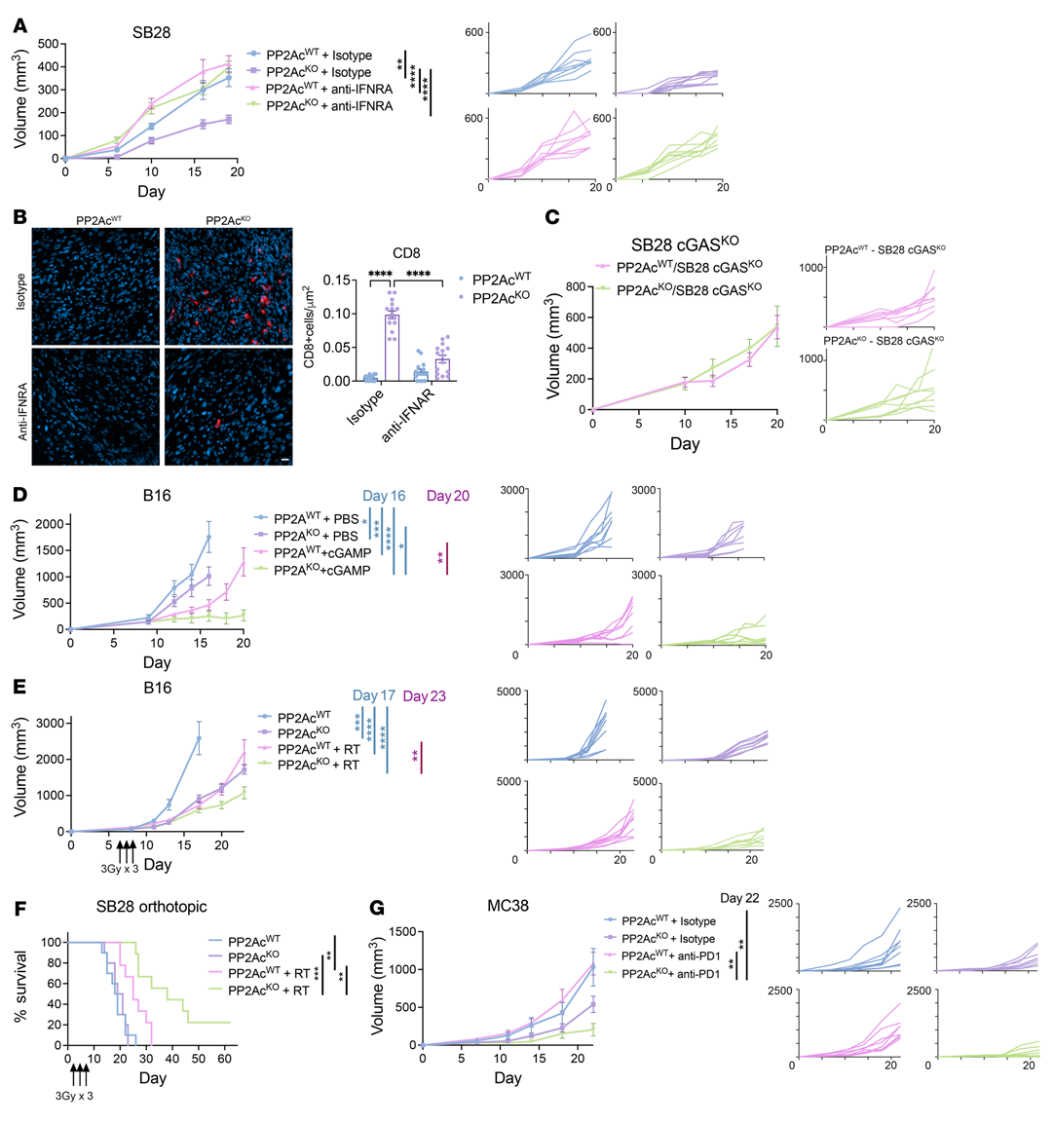
Macrophage PP2Ac deficiency synergizes with STING agonist, radiation, and immune checkpoint blockade. (**A**) *LysM^cre^PP2Ac^fl/fl^* or WT *C57BL/6* mice were inoculated with 0.1 × 10^6^ SB28 cells s.c. (*n* = 8). Mice were given intra-tumoral injection of anti-IFNAR-1 or isotype control (100 μg) on days 0 and 2, and then 2 times per week. (**B**) At survival endpoint, histological analysis was performed, staining for CD8 (red) and nucleus (4,6-diamidino-2-phenylindole (DAPI), blue). Scale bar: 10 μm. CD8 cells per field of view from 3 areas of interest on 3 independent samples (*n* = 9) were quantified. (**C**) *LysM^cre^PP2Ac^fl/fl^* or WT mice were inoculated with 0.1 × 10^6^ SB28 cGAS^KO^ (*n* = 8) cells s.c. (**D**) *LysM^cre^PP2Ac^fl/fl^* or WT mice were inoculated with 0.1 × 10^6^ B16 cells s.c. At day 4, mice were randomized (*n* = 7-8) into intratumoral injection of PBS or cGAMP (3 μg) at days 4, 8, and 11. (**E**) *LysM^cre^PP2Ac^fl/fl^* or WT mice were inoculated with 0.1 × 10^6^ B16 cells s.c. At day 7, mice were randomized to with or without radiation (*n* = 8). For the radiation groups, tumors were locally irradiated with 3Gy daily for 3 consecutive days (3 × 3Gy). (**F**) *LysM^cre^PP2Ac^fl/fl^* or WT mice were inoculated with 3 × 10^4^ SB28 cells in the brain. At day 5, mice were randomized to with or without radiation (*n* = 9–10). Cumulative survival of mice over time. (**G**) *LysM^cre^PP2Ac^fl/fl^* or WT mice were inoculated with 1 × 10^6^ MC38 cells s.c. At day 7, animals were randomized to treatment with anti-PD-1 or IgG1 isotype (200 μg) antibodies via i.p injection, given twice a week. Data are from 1 experiment representative of at least 2 (for **C**–**G**) or 1 (for **A** and **B**) independent experiments with similar results. Mantel-Cox log-rank tests were used for survival analysis. Error bars depict SEM. *P* values were calculated by 1-way ANOVA with Tukey’s multiple comparison test. *P < 0.05, **P < 0.01, ***P < 0.001, ****P < 0.0001.

Given our in vitro finding that PP2Ac-deficient macrophages have enhanced Type I IFN signaling (Figure 1A), we asked whether type I IFNs was required for the antitumor effect of macrophage PP2Ac KO in vivo. Mice bearing SB28 (Figure 3A) or B16 (Supplemental Figure 6A) tumors were treated with intratumoral injections of isotype or IFNAR-blocking antibody on days 0 and 2 after tumor implantation followed by biweekly injections in *LysM^cre^PP2Ac^fl/fl^* or WT mice. Blocking type I IFN signaling abrogated the therapeutic effect of macrophage specific PP2Ac deficiency in both models and restored tumor sizes to levels similar to WT mice, suggesting that type I IFNs are essential for eliciting PP2Ac-deficiency–mediated antitumor response in TAMs. We then asked whether blockade of type I IFN signaling will affect the degree of CD8^+^ T cell infiltration in the tumor microenvironment. At survival endpoint, tumors were harvested and stained for CD8^+^ T cells by immunofluorescence. We found that the increase in tumor infiltrating CD8^+^ T cells in *LysM^cre^PP2Ac^fl/fl^* mice was significantly reduced once IFN signaling was blocked (Figure 3B), suggesting that Type I IFN signaling was required for PP2Ac-deficient macrophages to promote enhanced adaptive antitumor immunity.

Since we found that macrophage PP2Ac deficiency enhanced STING-mediated IFN signaling (Figure 1, D–J) in vitro, we asked if promotion of STING activation in macrophages was responsible for enhanced antitumor immunity in *LysM^cre^PP2Ac^fl/fl^* mice in vivo. Several recent studies demonstrated that the export of tumor-derived cGAMP to activate STING in host immune cells is essential to eliciting a successful antitumor response (6, 8, 27). We tested if cGAS KO in tumor cells, which will deplete the source of cGAMP to activate host STING, will abolish the therapeutic effect of macrophage PP2Ac deficiency. To this end, we generated cGAS^KO^ in B16 and SB28 cell lines using CRISPR/Cas9 and confirmed that cGAS protein expression was absent in these cells (Supplemental Figure 2, B and C). We found that the effect of reduced tumor growth in *LysM^cre^PP2Ac^fl/fl^* mice was abolished when cGAS was deficient in SB28 (Figure 3C) or B16 (Supplemental Figure 6B) tumors, suggesting that tumor cGAMP production, which is responsible for STING activation in host macrophages, is required for PP2Ac-mediated regulation of macrophage tumor immunity.

Next, since administration of STING ligands has been shown to induce robust antitumor response in preclinical models (3) we asked if macrophage PP2Ac deficiency can synergize with cGAMP treatment in vivo by inoculating s.c. B16 tumors in *LysM^cre^PP2Ac^fl/fl^* and WT mice. At day 4, mice were randomized to intratumoral injections of cGAMP or PBS. Treatment was given on days 4, 8, and 11. Macrophage PP2Ac deficiency synergized with cGAMP treatments, as *LysM^cre^PP2Ac^fl/fl^* mice had reduced tumor growth compared with WT mice (Figure 3D). Since STING has been shown to be essential for therapeutic radiation (1), we tested the synergistic potential between radiation- and macrophage-PP2Ac deficiency. Given that radiation is part of standard-of-care treatment for melanoma and glioma, we used orthotopic models of B16 melanoma and SB28 glioma. s.c. B16 tumors were injected in *LysM^cre^PP2Ac^fl/fl^* and WT mice before being randomized on day 7 for local radiation with 3Gy on days 7, 8, and 9 for a total dose of 9Gy. Radiation treatment in *LysM^cre^PP2Ac^fl/fl^* mice significantly reduced tumor growth compared with WT mice (Figure 3E), although the effect is less pronounced than cGAMP treatment. We also tested the effect of macrophage PP2Ac deficiency with radiation in i.c. SB28 glioma. SB28 is a poorly immunogenic syngeneic glioma model (25). Consistent with earlier reports that i.c. SB28 is less immunogenic than s.c. SB28 (24), there was no survival difference between *LysM^cre^PP2Ac^fl/fl^* and WT mice (Figure 3F), in contrast to our observation in s.c. SB28 (Figure 2B). However, in combination with radiation, i.c. SB28 in *LysM^cre^PP2Ac^fl/fl^* had significantly prolonged survival, with 20% achieving complete remission (Figure 3F). Finally, given that STING activation has been shown to potentiate the effect of checkpoint immunotherapy (26), we tested the effect of macrophage PP2Ac^KO^ on anti-PD1 treatment in s.c. MC38 tumors in *LysM^cre^PP2Ac^fl/fl^* and WT mice. On day 7, mice were randomized to treatment with isotype control or anti-PD1 blockade given twice a week until the survival endpoint. While anti-PD1 failed to provide any benefit in WT mice, macrophage PP2Ac deficiency sensitized MC38 to checkpoint blockade in *LysM^cre^PP2Ac^fl/fl^* mice (Figure 3G). Collectively, our results demonstrated that macrophage PP2Ac deletion reduced tumor growth in a cGAS-STING dependent manner and synergized with STING-activating treatments including cGAMP, radiation, and anti-PD1 blockade in multiple syngeneic tumor models.

### scRNA-Seq analysis reveals macrophage-PP2Ac deficiency alters the tumor immune microenvironment.

**Figure 4:**
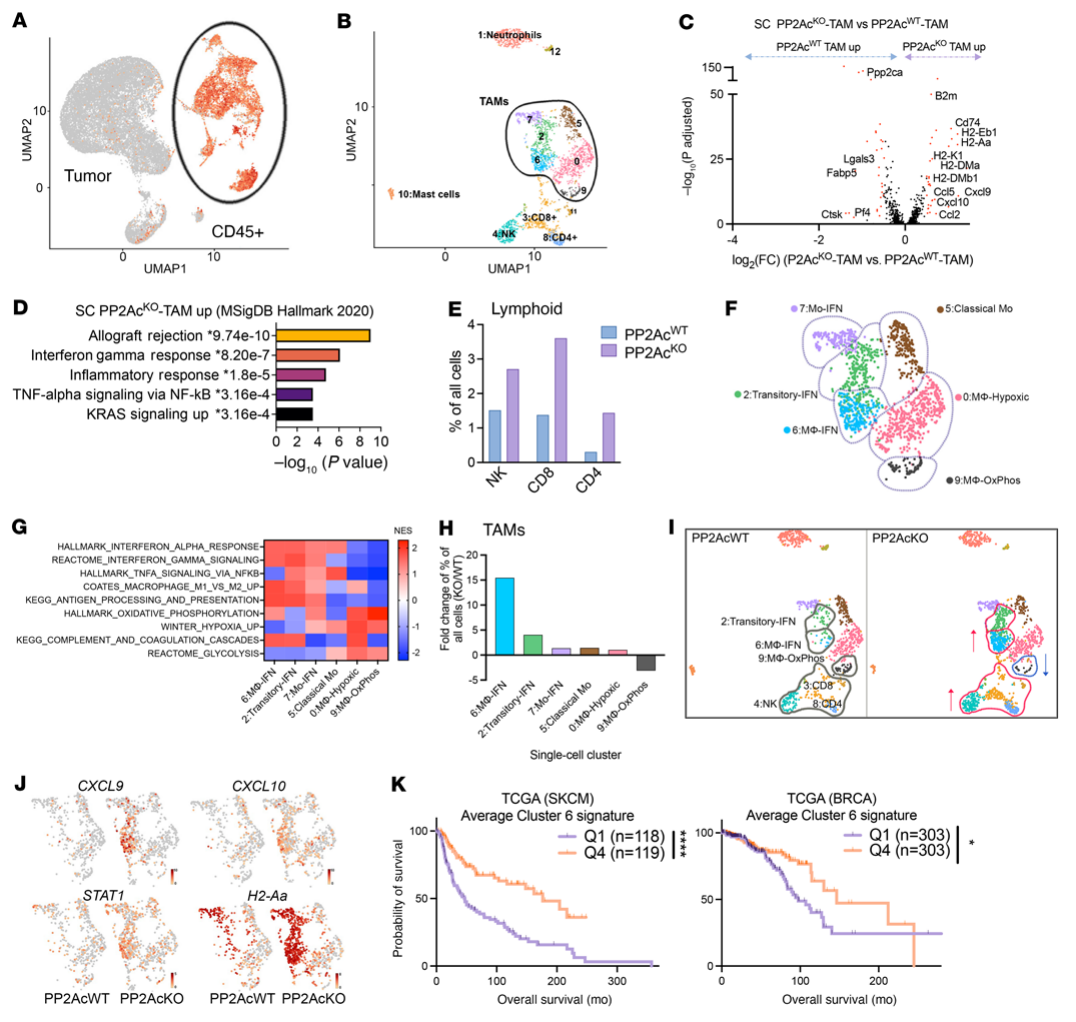
scRNA-Seq of s.c. SB28 tumor. *LysM^cre^PP2Ac^fl/fl^* or WT mice were inoculated with SB28 tumor subcutaneously (0.1 × 10^6^ cells) or orthotopically in the brain (3 × 10^4^ cells). On day 18, 3 tumors per group were pooled and analyzed by scRNA-Seq. (**A**) UMAP analyses were performed on 26,023 cells from all 4 groups. (**B**) UMAP of CD45^+^ immune cells of s.c tumors. Canonical markers were used to identify major immune populations. (**C**) Volcano plots showing DEGs (−log_10_ (adjusted P) > 2, log_2_FC > 0.5) in CD68^+^TAMs between tumors from *LysM^cre^PP2Ac^fl/fl^* or WT mice. *P* values were adjusted using Bonferroni’s correction. Upregulated genes related to antigen presentation and IFN signaling are labelled. (**D**) Pathway enrichment analysis performed using Enrichr on upregulated DEGs in tumors from *LysM^cre^PP2Ac^fl/fl^* mice. Top 5 enriched biological processes ranked by –log(*P*). (**E**) Percentage of lymphoid (CD4, CD8, and NK) cells of all cells. (**F**) Overview of TAM subsets with 6 subclusters: subcluster 0, hypoxic macrophage; subcluster 2, transitory-IFN; subcluster 5, classical monocyte; subcluster 6, IFN macrophage; subcluster 7, IFN monocytes; and subcluster 9, oxidative phosphorylation (Ox-Phos) macrophage. (**G**) Heatmap of Normalized Enrichment Score (NES) from GSEA of TAMs cluster. (**H**) Fold change in frequency of the 6 TAMs clusters. (**I**) UMAP of immune cells highlighting the clusters that are altered between tumors from *LysM^cre^PP2Ac^fl/fl^* or WT mice. (**J**) UMAP of TAMS highlight IFN-response genes (CXCL9, CXCL10, STAT1, and H2-Aa) in TAMs from *LysM^cre^PP2Ac^fl/fl^* or WT mice. (**K**) Average expression level of cluster 6 gene signatures associated with survival of patients with melanoma and breast cancer from TCGA bulk RNA-Seq data set (SKCM and BRCA respectively). Mantel-Cox log-rank tests were used for survival analysis. *P < 0.05, ****P < 0.0001.

**Figure 5:**
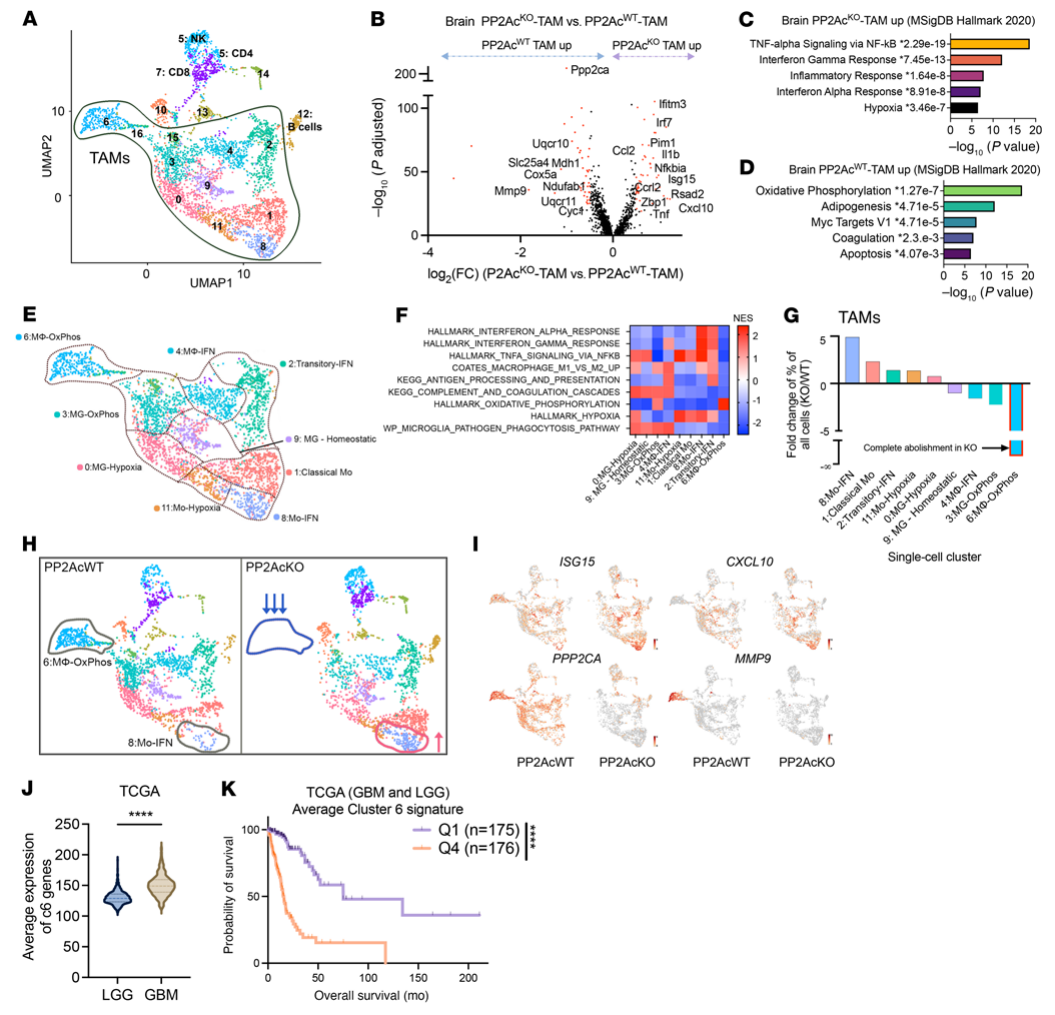
scRNA-Seq of i.c. SB28 tumor. (**A**) UMAP of CD45^+^ immune cells of i.c tumors in Figure 4A. Canonical markers were used to identify major immune populations. (**B**) Volcano plots showing DEGs (−log_10_ (adjusted *P*) > 20, log_2_FC > 0.5) in CD68^+^TAMs between tumors from *LysM^cre^PP2Ac^fl/fl^* or WT mice. *P* value adjusted using Bonferroni’s correction. Upregulated genes related to IFN signaling, and downregulated genes related to oxidation phosphorylation are labelled. (**C** and **D**) Pathway enrichment analyses using Enrichr showing upregulated (**C**) and downregulated (**D**) DEGs of TAMs in tumors from *LysM^cre^PP2Ac^fl/fl^* mice. Top 5 enriched biological processes ranked by –log(*P*). (**E**) Overview of TAM subsets with 9 subclusters identified: subcluster 0, hypoxic macrophage; subcluster 1, classical monocytes; subcluster 2, transitory-IFN; subcluster 3, Ox-Phos microglia; subcluster 4, IFN macrophage; subcluster 6, Ox-Phos macrophage; subcluster 8, IFN monocytes; subcluster 9, homeostasis microglia; and subcluster 11, hypoxic monocytes. (**F**) Heatmap of Normalized Enrichment Score (NES) from GSEA identified pathway enrichment in each TAM cluster. (**G**) Fold change in frequency of the 9 TAMs clusters. (**H**) UMAP of immune cells highlighting the clusters that are altered between tumors from *LysM^cre^PP2Ac^fl/fl^* or WT mice. (**I**) UMAP of TAMS highlight IFN-response genes (CXCL10 and ISG15), MMP9 and PP2Ac in TAMs from *LysM^cre^PP2Ac^fl/fl^* or WT mice. (**J**) Average expression of cluster 6 gene signature is higher in high grade glioma (*n* = 171) than in low grade glioma (*n* = 530) from TCGA data set. *P* value calculated by 2-tailed unpaired *t* test (****P < 0.0001). (**K**) Average expression level of cluster 6 gene signature is associated with worse survival in patients with glioma using TCGA bulk RNA-Seq data set (merged LGG and HGG). Mantel-Cox log-rank tests were used for survival analysis. ****P < 0.0001.

To explore how macrophage PP2Ac deficiency reshapes the tumor immune environment, we performed scRNA-Seq in both s.c. and i.c. models of SB28 glioma from *LysM^cre^PP2Ac^fl/fl^* and WT mice. Tumors were harvested on day 18, and tissues from 3 mice were pooled for each group. Whole cell content was analyzed by scRNA-Seq. Unsupervised clustering and uniform manifold approximation and projection (UMAP) analyses were performed on 26,023 cells from all 4 groups (Figure 4A). Immune cells identified by CD45^+^ expression were selected for further analysis (see Methods). Seurat package (28) was used to perform fine clustering of immune cells in s.c. (Figure 4) and i.c. (Figure 5) tumors.

In s.c. tumors, 12 seurat clusters were identified (Figure 4B, Supplemental Figure 7A, and Supplemental Table 1). Canonical markers were used to classify the 12 clusters into 10 major immune populations (Figure 4B and Supplemental Figure 7B), including CD8^+^T cells (CD3^+^CD8b1; cluster 3); CD4^+^T cells (CD3^+^CD4^+^; cluster 8); NK cells(NCR1^+^; cluster 4); Macrophages (TAMs) (CD68^+^; clusters 0, 2, 5, 6, 7, and9); Neutrophils (S100a9^+^; cluster 1); and Mast cells (Tpsb2^+^; cluster 10). We first explored the effect of macrophage PP2Ac^KO^ on TAMs by examining the differentially expressed genes (DEGs) (minimum 2-fold, adjusted *P* value < 0.01; clusters 0, 2, 5, 6, 7, and 9) (Figure 4C). We found a significant increase in expression of genes encoding components of MHC class I (B2m, H2-D1, and H2-K1), MHC class II (H2-Eb1, H2-DMa, H2-Aa, and H2-DMb1), and IFN-response genes (CXCL9, CXCL10, CCL5, and CCL2). Functional analysis suggested a significant (adjusted *P* value of less than 0.05) enrichment of the gene sets associated with immune response, such as IFNγ and TNF signaling (Figure 4D). In addition, there was an increase in lymphoid cell (NK, CD8, and CD4) infiltration (Figure 4E). We further examined the subpopulation structure of TAMs. Six major monocyte/macrophage subpopulations were identified by unbiased clustering. The top 10 marker genes for each cluster were shown in a heatmap (Supplemental Figure 8A). Cluster 5 expressed classical monocyte markers *(*Ly6c2^+^Chil3^+^CCR2^+^). Tumor associated macrophages downregulated Ly6c2/Chil3 and upregulated macrophage markers, such as C1QA (clusters 0 and 6). Clusters 2 and 7 expressed monocyte-related genes such as CCR2 but not others, such as Ly6c2 and Chil3 (Supplemental Figure 8, A and B). The expression of mature macrophage marker C1QA increased from cluster 7 to cluster 2, suggesting ongoing monocyte-to-macrophage differentiation from cluster 5 (classical monocytes) to clusters 0 and 6 (macrophages). Clusters 6, 2,and 7 all expressed high levels of IFN and MHC class I/II gene signatures (Figure 4I). Cluster 0 was a subpopulation of macrophages that exhibited clear hypoxic (such as BNIP3 and ADAM 8) and oxidative phosphorylation (such as MDH2 and CYC1) expression profiles, which are associated with proangiogenic and immunosuppressive properties (29, 30). Accordingly, they have low IFN signaling and MHC I/II expression (Figure 4, F and G). Cluster 9 is a small, distinct population of macrophages that not only expressed a high level of oxidative phosphorylation but also had an exclusively high level of MMP-9, secretion of which by TAMs has been associated with tumor progression and mesenchymal transition (31, 32). GSEA showed the relative expression of pathways associated with IFN signaling, hypoxia, oxidative phosphorylation, and antigen presentation between the 6 TAM clusters (Figure 4G and Supplemental Figure 9).

Next, we examined the relative frequency of each TAM cluster between tumors from *LysM^cre^PP2Ac^fl/fl^* and WT mice. We identified that cluster 6, the macrophage population with a high IFN signature, was more than 15-fold enriched in *LysM^cre^PP2Ac^fl/fl^* mice. Cluster 2, the transitory macrophages with high IFN activation, was also enriched 4-fold. Cluster 9, the subgroup with high oxidative phosphorylation and MMP9 was decreased 3-fold (Figure 4, H and I). Figure 4J illustrates that the increase in IFN signaling and MHC expression in TAMs (Figure 4C) is attributed to the increase in clusters 2 and 6 in *LysM^cre^PP2Ac^fl/fl^* mice. In summary, the scRNA-Seq data suggest that macrophage PP2Ac^KO^ led to the differentiation of monocytes toward IFN-activated, proinflammatory macrophages (clusters 2 and 6) while suppressing antiinflammatory, immunosuppressive macrophages with high oxidative phosphorylation and MMP9 expression (cluster 9). Lastly, to determine if the population of macrophages (cluster 6) we identified to be enriched by macrophage PP2Ac^KO^ has relevance in human cancer, we obtained bulk RNA-Seq data set from The Cancer Genome Atlas (TCGA) for melanoma and breast cancer (SKCM and BRCA, respectively). We calculated the average normalized expression of the cluster 6 (IFN signature) gene signature and compared survival between patients with high and low expression — 4^th^ and 1^st^ quartile (Figure 4K). We found that higher cluster 6 signatures had significantly improved survival in both cancer types, suggesting that targeting PP2Ac in macrophages to promote IFN-activated TAMs could have relevance in human cancer.

It is well known that the tumor microenvironment in the brain is immunosuppressive; therefore, it is not surprising that we observed a significant difference in tumor size of s.c. SB28 but no survival difference in i.c. SB28 tumor in the absence of radiation between *LysM^cre^PP2Ac^fl/fl^* and WT mice (Figure 3F). This led us to explore how macrophage PP2Ac^KO^ differentially remodeled the immune landscape in i.c. SB28 using scRNA-Seq. From the initial UMAP analysis (Figure 4A), CD45^+^ cells were reclustered. Sixteen seurat clusters were identified (Figure 5A, Supplemental Figure 10A, and Supplemental Table 2). Using canonical markers, major immune populations were identified (Figure 5A and Supplemental Figure 10B), CD8^+^ T cells (CD3^+^CD8b1; cluster 7); CD4^+^ T cells (CD3^+^CD4^+^; cluster 5); NK cells (NCR1^+^; cluster 5); Macrophages (TAMs) (CD68^+^; clusters 0, 1, 2, 3, 4, 5, 8, 9, 11, 15, and 16) and B cells (MS4A1^+^; cluster 12). As expected, the immune microenvironment of the brain is more complex, with a contribution of ontologically distinct myeloid cells including yolk sac-derived microglia (MG) and monocytic-derived macrophages (33, 34).

We examined the DEGs (minimum 2-fold, adjusted *P* value < 0.001) in the TAM population and found enhanced IFN and TNF-related gene expressions (CXCL10, ISG15, IFITM3, IRF7, and NFKBIA), which are similar to s.c. tumors (Figure 5, B and C). Functional analysis showed upregulation of (adjusted *P* value of less than 0.05) MSigDB hallmark gene sets associated with TNF, Type I and II IFN immune responses (Figure 5C), and downregulation of gene sets associated with oxidative phosphorylation (Figure 5D). We examined the subpopulation structure of TAMs with 9 major monocyte/macrophage subsets identified by unbiased clustering. The top 10 marker genes for each cluster are shown in a heatmap (Supplemental Figure 11A). Clusters 1, 8, and 11 expressed classical monocyte markers Ly6C2, Chil3, CCR2; cluster 8 expressed high levels of IFN-related genes CXCL10, ISG15, IRF7; and Cluster 11 expressed upregulated hypoxia-related genes Bnip3, PTGS2, ADAM8. Using CX3CR1 and TMEM119 as markers (34, 35)^,^ we identified clusters 0, 3, and 9 as MG. Cluster 9 displayed classic homeostatic MG signatures, including P2RY12, SIGLECH, and CST7, whereas clusters 0 and 3 showed hypoxia and oxidation phosphorylation–related gene signatures, respectively. Cluster 2 was consistent with a transitory subgroup between monocytes and macrophages, with loss of monocyte marker Chil3, but maintenance of others such as CCR2, PLAC8, and Ly6C2. Cluster 4 expressed the mature macrophage marker C1QA, suggesting monocyte-to-macrophage differentiation from clusters 1, 8, and 11 to clusters 2 and 4. Both clusters 2 and 4 expressed IFN signature and MHC class I/II genes, consistent with activated macrophages. Similar to s.c. tumors, there was a subset of MMP9^+^ TAMs with high oxidative phosphorylation (cluster 6) (Figure 5E and Supplemental Figure 11B), which is more prominent in i.c. tumors. GSEA showed the relative expression of pathways associated with IFN/TNF signaling, hypoxia, oxidative phosphorylation, and antigen-presentation between the 9 TAM clusters (Figure 5F and Supplemental Figure 12).

Next, we examined the relative frequency of each cluster between tumors from *LysM^cre^PP2Ac^fl/fl^* and WT mice. We found that cluster 8, the monocyte subpopulation with high IFN signature, was 5-fold enriched in *LysM^cre^PP2Ac^fl/fl^* mice, whereas cluster 6, the MMP9-high/oxidative phosphorylation cluster, was completely abolished (Figure 5, G and H). We found that cluster 8, the monocyte subpopulation with high IFN signature (Mo-IFN) contributed to an overall increase in expression of IFN-related genes, such as ISG15 and CXCL10 (Figure 5I), but this cluster contributed to a relatively small component of TAMs. Cluster 6, the MMP9-high TAMs, which are absent in tumors from *LysM^cre^PP2Ac^fl/fl^* mice, has the highest expression of PP2Ac relative to other TAM subsets (Figure 5I), suggesting these cells could be dependent on PP2Ac function. To determine whether the high MMP9/oxidative phosphorylation population (cluster 6) is relevant in human glioma, we obtained bulk RNA-Seq data sets from TCGA with combined low-grade glioma (LGG) and high-grade GBM. We calculated the average normalized expression of cluster 6 signature and found that GBM had higher expression than LGG (Figure 5J). Survival is worse for patients with higher cluster 6 expressions (Figure 5K). However, expression of cluster 6 gene signatures did not independently predict survival within patients with LGG or GBM (Supplemental Figure 13, A and B), suggesting that the signature’s association with higher-grade tumors contributed to its correlation with survival. In summary, the scRNA-Seq data of i.c. tumors suggest that macrophage PP2Ac^KO^ led to significant remodeling of the myeloid composition in i.c. tumors in ways that differ from s.c. tumors. However, in both s.c. and i.c. tumors, there is a significant upregulation of Type I IFN and downregulation of the high MMP9/oxidative phosphorylation subset of TAMs.

### Regulatory B subunit of PP2A, STRN4, negatively regulates STING-Type I IFN by modulation of Hippo kinase MST1/2 and YAP/TAZ.

**Figure 6:**
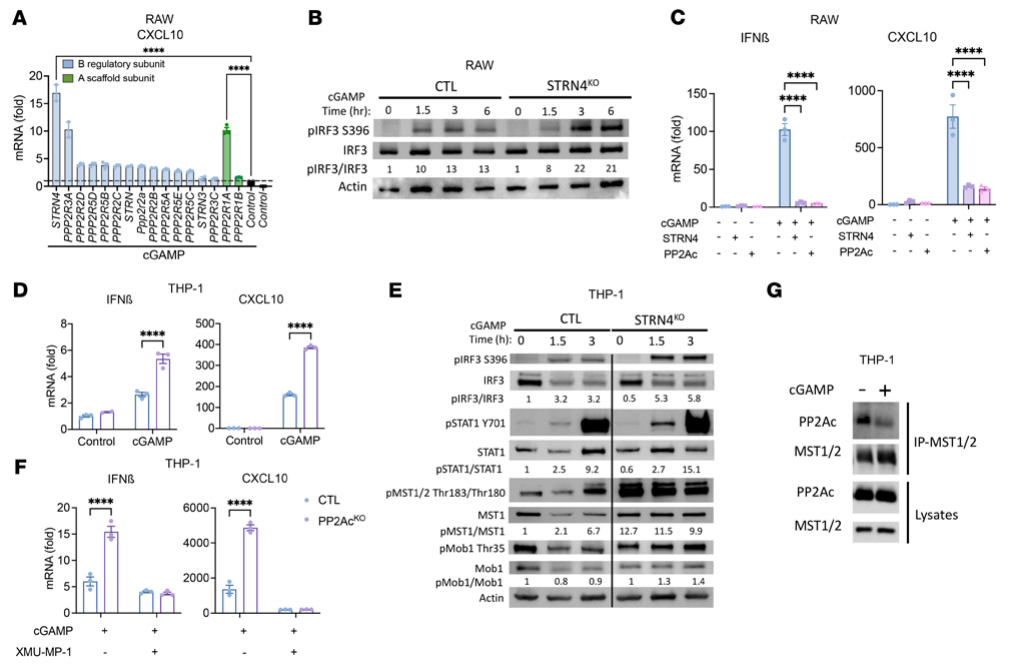
STRN4, a regulatory B subunit of PP2A, negatively regulates STING-Type I IFN by modulation of Hippo kinase MST1/2 and YAP/TAZ. (**A**) siRNA screen identified PP2A subunits involved in cGAS-STING response. RAW cells were transfected with siRNA of each of 14 regulatory and 2 scaffold subunits of PP2A. 48 hours after transfection, cells were treated with cGAMP (10 μg/mL) for 4 hours. CXCL10 expression was measured via real time PCR. Fold change is relative to nontargeting siRNA. (**B**) CTL and STRN4^KO^ RAW cells were treated with cGAMP (10 μg/mL), protein expression was analyzed by immunoblotting at different time points after stimulation. (**C**) RAW cells were transfected with overexpression plasmids for STRN4 or PP2Ac. 48 hours after transfection, cells were treated with cGAMP (10 μg/mL) for 4 hours. Expression of IFNβ and CXCL10 was measured via real time PCR. (**D**) CTL and STRN4^KO^ THP-1 differentiated macrophages were treated with cGAMP (10 μg/mL) for 4 hours. Expression of IFNβ and CXCL10 was measured via quantitative PCR. (**E**) CTL and STRN4^KO^ THP-1 differentiated macrophages were treated with cGAMP (10 μg/mL). Protein expression was analyzed by immunoblotting at different time points after stimulation. (**F**) THP-1 differentiated macrophages were treated with MST-1 inhibitor, XMU-MP-1 (1 μM), for 2 hours, before stimulation with cGAMP (10 μg/mL). 4 hours later, expression of IFNβ and CXCL10 was measured via real time PCR. (**G**) THP-1 differentiated macrophages were treated with or without cGAMP (10 μg/mL) and 1.5 hours later protein was collected. MST1/2 antibody was used for co-IP and blotted for PP2Ac and MST1/2. Data are from 1 experiment representative of 3 (for **B**–**E**) and 2 (for **A** and **G**) independent experiments with similar results. Lanes (**E** and **F**) separated by black vertical line were run on the same gel but were noncontiguous. Error bars depict SEM. *P* values were calculated by 2-tailed unpaired *t* test. ***P < 0.001, ****P < 0.0001.

To further dissect the mechanism of PP2Ac-mediated regulation of macrophages, we set out to determine the regulatory B PP2A subunit responsible for modulating STING activation. The B subunit belongs to 4 structurally distinct families (B55, B56, PR70/72, and STRN) that exhibit little sequence similarity (36). While we previously demonstrated that pharmacological inhibition of PP2Ac synergizes with checkpoint immunotherapy (13, 14), a limitation of this strategy is that PP2Ac is widely expressed in multiple cell types and regulates many signaling pathways that can both inhibit and enhance tumor growth (11, 37). To systematically identify the PP2A holoenyzme that regulates STING signaling in macrophages, we conducted a loss-of-function screen using an siRNA library that targets each of the known PP2A B regulatory subunits in RAW cells in response to cGAMP treatment. We found that suppression of STRN4 resulted in the greatest statistically significant increase of CXCL10 expression after cGAMP treatment (Figure 6A). We also confirmed that silencing of a scaffolding A subunit, PPP2R1A, enhanced cGAMP-induced CXCL10 expression (Figure 6A).

Next, we explored the role of STRN4 in regulating STING-Type I IFN signaling in macrophages by generating STRN4^KO^ RAW cells using CRISPR/Cas9 (Supplemental Figure 2D). We confirmed that pIRF3, the immediate downstream signaling of STING activation, was enhanced in STRN4^KO^ in response to cGAMP (Figure 6B). Conversely, we tested to see whether STRN4 or PP2Ac negatively regulated STING response by overexpressing STRN4 or PP2Ac. Each dramatically diminished cGAMP-induced IFNβ and CXCL10 transcription (Figure 6C). To confirm STRN4’s role in regulating STING-Type I IFN signaling in human cells, we generated STRN4^KO^ ThP-1 cells using CRISPR/Cas9 (Supplemental Figure 2E). Consistently, STRN4^KO^ ThP-1–differentiated macrophages had significantly enhanced cGAMP-induced CXCL10 and IFNβ expression (Figure 6D). Increased STING activation was further validated by increased pIRF3 and pSTAT1 (Figure 6E). In summary, both loss-of-function and gain-of-function experiments suggested that the STRN4-PP2Ac complex negatively modulated STING-mediated Type I IFN response in macrophages.

It is known that STRN4 and PP2Ac are components of the striatin-interacting phosphatase and kinase (STRIPAK) complex that initiates the Hippo kinase cascade, consisting of the MST1/2 phosphorylating and activating the large tumor suppressor (LATS1/2), which in turn phosphorylates and inhibits YAP and TAZ (38). YAP and TAZ are transcription coactivators that are the functional output of Hippo signaling to regulate gene expression. YAP/TAZ in cancer cells has been shown in many studies to promote tumor growth. Therefore, Hippo kinase MST1/2 is recognized as a tumor suppressor gene by negatively regulating YAP/TAZ (39). However, the role of STRN4 in immune cell signaling is unexplored. In addition, the relevance of the Hippo-YAP/TAZ pathway in TAMs has not been studied. Given our data demonstrating the importance of STRN4 in regulating STING activation in macrophages, we hypothesize that STRN4 implicates the Hippo-YAP/TAZ pathways to regulate STING-Type I IFN signaling. We found that in STRN4^KO^ THP1–differentiated macrophages and phosphorylation of MST1/2 and its downstream substrate, Mps1-binder-related (MOB) protein was significantly enhanced in response to cGAMP (Figure 6E), indicating that STRN4 negatively regulated Hippo kinases in macrophages. Next, we asked if Hippo signaling was required for STING activation by treating THP1 differentiated macrophages with the MST1/2 inhibitor XMU-MP-1 prior to cGAMP activation. XMU-MP-1 completely abolished cGAMP-induced CXCL10 and IFNβ gene expression in both control and PP2Ac^KO^ THP-1 differentiated macrophages (Figure 6F). These results suggest that the Hippo cascade is essential for STING activation and that MST1/2 acts downstream of PP2A-STRN4 in modulating STING activity. To provide further biochemical evidence that STRN4-PP2A interacts with MST1/2 in regulating STING activity, we performed an immunoprecipitation assay of protein lysates from untreated and cGAMP-treated ThP-1 differentiated macrophages by pulling down MST1/2 and blotting for PP2Ac. We found that,in resting conditions, PP2Ac was associated tightly with MST1/2. Upon cGAMP treatment, PP2Ac dissociated from MST1/2, decoupling the dephosphorylation activity of PP2Ac and thereby increasing the activation of MST1/2 (Figure 6G). Collectively, we established the role of STRN4-PP2A in negatively regulating STING signaling by inactivating Hippo signaling.

### YAP/TAZ mediates STRN4-PP2A modulation of STING signaling in macrophages and is highly enriched in TAMs.

YAP/TAZ is a well-characterized downstream effector of the Hippo pathway and is implicated in diverse cellular processes including tissue homeostasis, organ regeneration, and tumorigenesis (40). Upregulation of YAP/TAZ correlates with poor prognosis in multiple cancers (39). However, the role of YAP/TAZ in modulating immune cell function is an emerging area of investigation (38). 2 recent studies reported that, in antiviral response of macrophages, YAP inhibits Type I IFN signaling by antagonizing TBK1 or IRF3 independently of its transcriptional activity (41, 42). However, the significance of YAP/TAZ in TAMs is unknown and it is unclear if the STRN-PP2A complex regulates YAP/TAZ function in macrophages. Given that we showed that STRN4-PP2A negatively regulates MST1/2, a YAP/TAZ suppressor, we hypothesize that STRN4-PP2A stabilizes YAP/TAZ expression in macrophages to inhibit STING-Type I IFN.

**Figure 7:**
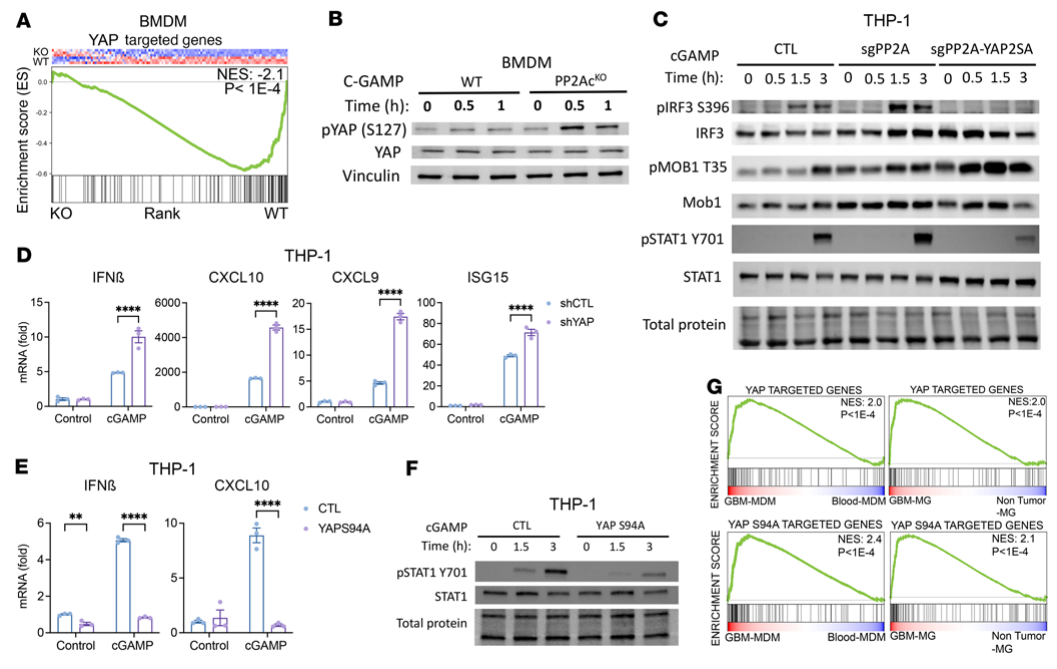
YAP/TAZ mediates STRN4-PP2Ac regulation of STING signaling in macrophage. (**A**) GSEA of YAP targeted genes in PP2Ac^KO^ versus PP2Ac^WT^ BMDM treated with cGAMP using RNA-Seq data set from Figure 1A. (**B**) PP2Ac^KO^ and PP2Ac^WT^ BMDMs were treated with cGAMP and protein expression was analyzed. (**C**) CTL, PP2Ac^KO^ and YAP overexpressed PP2Ac^KO^ THP-1 differentiated macrophages were treated with cGAMP and protein expression was analyzed. (**D**) shYAP THP-1 differentiated macrophages were treated with cGAMP for 4 hours and ISGs expression was measured. (**E**) YAPs94A overexpressed THP-1 differentiated macrophages were treated with cGAMP for 4 hours. ISGs expression was measured (**F**) YAPs94A overexpressed THP-1 differentiated macrophages were treated with cGAMP and protein expression was analyzed. (**G**) Publicly available RNA-Seq data set of sorted MDM and MG from human glioma samples, and blood MDM and nontumor MG were obtained. GSEA of YAP WT- and YAPS94A-targeted genes in GBM MDM versus blood MDM and GBM MG versus nontumor MG. Data are from 1 experiment representative of 3 independent experiments. Error bars depict SEM. *P* values were calculated by 1-way ANOVA with Tukey’s multiple comparison test. *P < 0.05, ****P < 0.0001.

First, we tested whether YAP signaling is decreased in PP2Ac^KO^ BMDM. Whole-transcriptome profiles of cGAMP-stimulated PP2Ac^KO^ and PP2Ac^WT^ BMDM from the RNA-Seq shown in Figure 1A were analyzed. GSEA demonstrated a decreased YAP signature in PP2Ac^KO^ BMDM (Figure 7A). This suggested that PP2Ac deficiency led to downregulation of YAP signaling. We then found an increase in phosphorylated YAP (pYAP) in PP2Ac^KO^ BMDM (Figure 7B) after cGAMP treatment. Phosphorylation of YAP is known to induce cytoplasmic translocation and inactivation (40). To demonstrate that YAP was acting downstream to PP2Ac in modulating STING-mediated Type I IFN response, we ectopically overexpressed YAP using retroviral transduction of PP2Ac^KO^ THP-1 cells (YAP2SA) (Supplemental Figure 2F). We found that YAP overexpression completely abolished the enhanced pIRF3 (Figure 7C and Supplemental Figure 14) and pSTAT1 (Figure 7C) expression in PP2Ac^KO^ THP1–differentiated macrophages in response to cGAMP. Consistent with YAP as a downstream effector of Hippo signaling, phosphorylation of MOB remained elevated in PP2Ac^KO^ compared with WT THP-1 cells when YAP was overexpressed (Figure 7C). To further confirm that YAP downregulation was sufficient to enhance Type I IFN response, we used lentiviral transduction of shRNA to generate YAP knockdown in THP-1 cells (Supplemental Figure 2G). Silencing of YAP, similar to downregulation of PP2Ac, enhanced cGAMP-induced Type I IFN and IFN response genes, including IFNβ, CXCL10, CXCL9, and ISG15 (Figure 7D). To test whether YAP inhibition of STING-Type I IFN signaling was dependent on its transcriptional activity, we overexpressed a mutated form of YAP, YAPs94A, which resulted in a diminished ability of YAP to bind its nuclear partner TEAD, and, thereby, its transcriptional function (43). We found that YAPS94A overexpression completely repressed cGAMP-induced IFNβ and CXCL10 transcription (Figure 7E) and diminished pSTAT1 (Figure 7F) expression, suggesting that negative regulation of STING signaling by YAP was independent of its transcriptional function.

We have shown that YAP expression in macrophages plays a critical role in response to exogenous cGAMP treatment. Next, we would like to establish relevance of exogenous cGAMP treatment in tumor conditions, as the role of YAP in regulating TAMs has not been explored. Using a previously published data set (18), we analyzed the RNA-Seq transcriptome profiles of sorted Monocyte Derived Macrophages (MDM) or MG from human high-grade GBM using nontumor blood MDM and MG as controls. Using a list of the top 200 upregulated genes associated with overexpression of WT YAP — which confers both transcriptional and nontranscriptional function — and YAPS94A — which lacks transcriptional function (44) — GSEA showed that both YAP- and YAPS94A-associated gene signatures were enriched in GBM-derived MDM or MG compared to nontumor blood MDM or MG, respectively (Figure 7G). This observation is recapitulated in murine glioma using the Genetic Engineered Glioma Model (GEMM) or GL261 (Supplemental Figure 15) (45). These data suggest that TAMs express a higher level of YAP relative to their healthy counterparts. In addition, the nontranscriptional YAP signature in TAMs is also enriched compared with the control, indicating that the role of YAP in TAMs could be dependent on its nontranscriptional activity.

**Figure 8:**
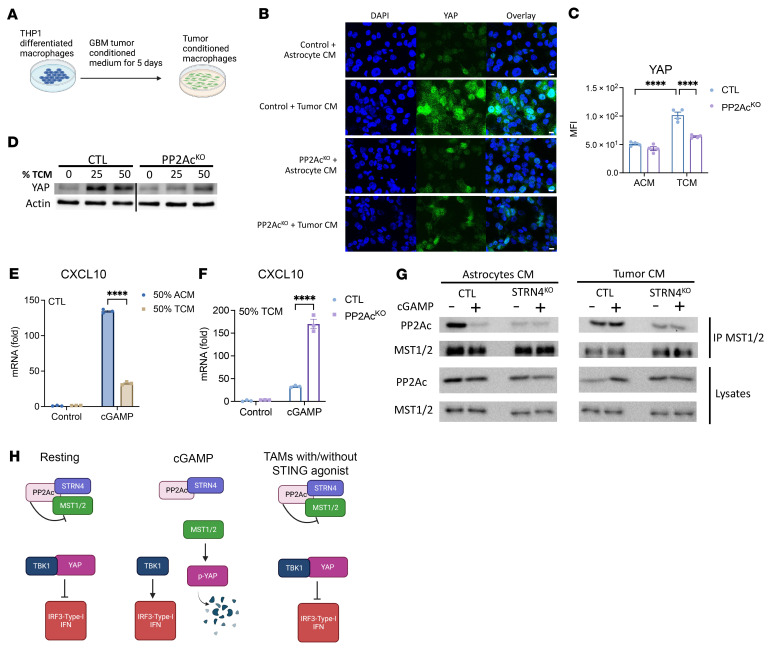
Tumor-induced YAP expression to suppress STING signaling in macrophage. (**A**) Scheme of experimental workflow. (**B**) Histological analysis of CTL and PP2A^KO^ THP-1 differentiated macrophages stained for YAP (green) and nucleus (DAPI, blue). Scale bar: 10 μm. Image representative of 4 independent regions (*n* = 4). (**C**) Quantification of YAP fluorescent intensity in selected regions. (**D**) YAP expression in TCM treated CTL and PP2A^KO^ THP-1 differentiated macrophages. (**E** and **F**) After 5 days in ACM or TCM, CTL or PP2Ac^KO^ THP-1–differentiated macrophages were treated with cGAMP for 4 hours. CXCL10 expression was measured. (**G**) After 5 days in ACM or TCM, CTL or STRN4^KO^ THP-1–differentiated macrophages were treated with cGAMP for 1.5 hours. MST1/2 antibody was used for co-IP and blotted for PP2Ac and MST1/2. (**H**) Model of PP2Ac/STRN4 mediated STING-IFN suppression in TAMs. cGAMP given at 10 μg/mL. Data are from 1 experiment representative of 3 independent experiments. Error bars depict SEM. *P* values were calculated by 1-way ANOVA with Tukey’s multiple comparison test. ****P < 0.0001.

Next, to test whether tumor-conditioned macrophages have enhanced YAP expression at the protein level, we treated THP1-differentiated macrophages with tumor-conditioned medium (TCM) from human glioma cell line (SF268) and astrocyte-conditioned medium (ACM) as control (Figure 8A). YAP expression was dramatically increased in TCM compared with ACM as measured by immunofluorescence (Figure 8, B and C) and Western blot (Figure 8D). Moreover, PP2Ac^KO^ THP-1 differentiated macrophages in TCM greatly reduced YAP expression (Figure 8, B and D). This was similarly observed in STRN4^KO^ THP-1 differentiated macrophages (Supplemental Figure 16). Given the negative regulatory role of YAP on STING signaling, we asked if TCM-primed macrophages would be less responsive to cGAMP. We found a decrease in cGAMP-induced CXCL10 transcription in TCM-primed THP-1–differentiated macrophages (Figure 8E). This TCM-induced suppression was significantly, but incompletely, reversed with PP2Ac ^KO^ (Figure 8F) or STRN4^KO^ (Supplemental Figure 16D). We then examined the biochemical interaction between MST1/2, PP2Ac, and STRN4 in TCM versus ACM in response to cGAMP. In ACM, similar the results shown in Figure 6G, cGAMP treatment resulted in dissociation of PP2Ac from MST1/2 (Figure 8G). However, in TCM, this dissociation failed to take place and MST1/2 remained bound to PP2Ac with cGAMP stimulation, suggesting downregulation of MST1/2 activity in TCM (Figure 8G). The association between PP2Ac and MST1/2 was diminished in STRN4^KO^ macrophages with or without cGAMP, suggesting that the interaction of PP2Ac and MST1/2 is mediated by STRN4 (Figure 8G). This is consistent with the role of the regulatory B subunit in conferring specificity and subcellular localization of the PP2A catalytic subunit. Collectively, these data raise several important observations. Consistent with our bioinformatic results showing that the YAP signature is enhanced in TAMs compared with nontumor macrophages, we demonstrated that YAP expression was upregulated in macrophages at the protein level when treated with TCM. The enhanced YAP level significantly blunted the degree of IFN signaling in response to STING agonists. Biochemical evidence suggested that the mechanism of YAP overexpression is through persistent association of PP2Ac with MST1/2, resulting in diminished Hippo signaling, which serves to phosphorylate YAP and leads to its inactivation and degradation. In macrophages, Hippo signaling appears to be essential for STING activation. However, the presence of tumor appeared to prevent activation of Hippo signaling by maintaining PP2Ac association with and thereby inactivation of MST1/2 (Figure 8H). This observation has clinical relevance as STING agonists are being investigated as a promising immunotherapeutic agent. However, thus far, clinical results have been disappointing. Our study provides a potential mechanistic framework to explain and overcome resistance to STING agonist therapy. We provide evidence that by inhibiting PP2Ac or its regulatory partner, STRN4, the tumor-induced brake on Hippo signaling can be relieved, leading to enhanced STING-Type I IFN response.

## Discussion

This study demonstrates that PP2Ac, together with the B subunit, STRN4, negatively regulates cGAS-STING-Type I IFN signaling in macrophages. PP2Ac^KO^ in macrophages remodels the immune tumor microenvironment to promote antitumor immunity and synergizes with STING agonists, radiation, and immune checkpoint blockades. scRNA-Seq of s.c. and i.c. mouse gliomas demonstrated in both models that macrophage PP2Ac deficiency upregulates Type I IFN-activated macrophages and downregulates high MMP9/oxidative phosphorylation macrophages. Bioinformatic analysis of TCGA database suggests a clinical relevance of these subpopulations in human cancer. Mechanistically, PP2Ac-STRN4 dephosphorylates and thereby deactivates the Hippo component MST1/2, which, in turn, results in stabilization of YAP by decreasing pYAP. In macrophages, the role of PP2Ac in modulating IRF3 or NFkB signaling in the setting of viral infections or Toll-Like Receptor (TLR) stimulation has been reported (19, 20). We uncovered a previously undescribed role of PP2A-STRN4 in modulating STING activation via Hippo signaling in TAMs. A key difference in the current study is that we specifically examined the regulatory mechanism of STING stimulation, as opposed to LPS/Poly IC and other TLR-mediated responses. While both TLR and STING stimulation share downstream mediators such as IRF3, TBK, and IKK, which are reported to be targets of modulation by PP2Ac (2), it is possible that distinct upstream stimulation — TLR or STING — elicits different feedback mechanisms that share PP2Ac as a common mediator, resulting in downregulation of their activation. It is also possible that there is crosstalk between RACK1, as described in Long, et al. (20), and YAP/Hippo components that has yet to be elucidated.

cGAS/STING is an important sensor for cytosolic dsDNA to elicit innate immunity. Both tumor cells and myeloid cells express cGAS and STING, but accumulating evidence suggests that, in the tumor microenvironment, cGAMP is primarily produced by tumor cells, due to the presence of cytoplasmic dsDNA. cGAMP is then released as an immunotransmitter to activate STING-Type I IFN signaling in myeloid cells (6–8). Tumor cells can evade STING activation by expressing ENPP1 to degrade cGAMP (6). Promoting STING-mediated Type I IFN signaling has been shown to promote antitumor response and synergize with checkpoint immunotherapy (26). Therefore, exogenous STING agonists are actively explored as a promising immunotherapy (46, 47). However, clinical trials of STING agonists have thus far failed to demonstrate significant clinical efficacy (9). Mechanistic understanding of STING agonist resistance is essential to formulate rational combination strategies. Our study provides a potential model to understand STING agonist resistance. We showed that dissociation of PP2Ac from Hippo kinase component MST1/2 is essential for STING agonists to activate downstream Type I-IFN signaling by facilitating downregulation of YAP. PP2Ac-MST1/2 interaction is mediated through the B regulatory subunit STRN4. We also observed that TCM prevented the dissociation of PP2Ac and MST1/2 in the presence of cGAMP, resulting in high levels of YAP expression. It is unclear what is the soluble factor in TCM or what upstream receptor in macrophages is responsible for this effect. STRNs could be recruited to membrane compartments by extracellular signals and regulate Hippo-YAP pathways in TAMs. Further studies are required to elucidate the mechanism underlying this crosstalk between tumor cells and macrophages through PP2A-STRN4. However, we did show that ablation of PP2Ac or STRN4 can partially reverse the TCM-induced inhibitory effect on Type I-IFN signaling. In addition, macrophage PP2Ac deficiency synergizes with STING agonists against tumor growth in vivo. Therefore, our study provides the preclinical evidence to argue for combining PP2Ac or STRN4 inhibition with STING agonist treatment to enhance antitumor immunity.

We also presented an unreported role for YAP in regulating macrophage function in the tumor microenvironment. Cancer cell-intrinsic expression of YAP promotes mesenchymal differentiation and stemness (48, 49), and its potential as a central cancer vulnerability has made it an attractive therapeutic target. However, most studies have focused on the role of YAP in cancer cells and its function in TAMs is unexplored. Recent studies showed that YAP inhibits macrophage-mediated antiviral responses independent of its transcriptional activity by associating with and directly antagonizing TBK1 and IRF3(41, 42), 2 downstream effectors of STING signaling. However, what induces YAP to antagonize Type I IFN signaling and whether this mechanism is relevant in TAMs is unknown. Our bioinformatic analysis and in vitro experiments suggest that YAP and genes associated with nontranscriptional YAP activity are enriched in TAMs relative to healthy myeloid cells, suggesting that YAP plays a role in mediating reprogramming of TAMs by tumors, possibly through its nontranscriptional activity. Our results, therefore, provide a rationale to inhibit YAP in TAMs to promote antitumor immunity in addition to the tumor-intrinsic benefit of YAP inhibition.

Our observation that macrophage PP2Ac deficiency confers benefit in s.c. but not i.c. tumors highlights the importance of the tumor microenvironment in dictating antitumor immune response. Comparing the scRNA-Seq data between s.c. and i.c. SB28 tumors suggests a potential explanation. Fundamentally, the difference between the i.c. and s.c. microenvironments is the presence of MG, an ontologically distinct resident myeloid population in the brain, which contribute to a more complex immune microenvironment. In both models, PP2Ac deficiency in myeloid cells resulted in an increase in a subset of IFN^hi^ macrophages (cluster 8 in i.c. and cluster 6 in s.c.) and a decrease in MMP9^+^ macrophages (cluster 6 in i.c. and cluster 9 in s.c.). However, the relative proportion of these altered clusters are smaller in i.c. compared with s.c. tumors. Therefore, the relative effect of PP2Ac KO on increasing IFN and decreasing MMP9 subpopulations in i.c. tumors is diluted by the presence of a pool of relatively unaltered TMEM119^+^ MG (clusters 0, 3, and 9) in i.c. tumors. Figure 5I demonstrates that PP2Ac expression is downregulated in TMEM119^+^ MG similarly as in BMDM, suggesting that MG do express the Lyz2 promoter with effective PP2A knock out. This raises the question of whether PP2Ac deletion has differential effect on BMDM and MG. It is unclear whether MG are more resistant to the effects of PP2Ac KO or whether PP2Ac KO in MG results in opposing (i.e., protumor) effects that counter the positive (i.e., antitumor) effect on BMDM. Further characterization of PP2Ac KO in MG both in vivo and in vitro, by generating TMEM119-Cre, PP2A-fl mice, is required to address this question.

## Methods

### Cell lines

Mouse melanoma cell lines B16F10, human monocytes THP1, mouse macrophage Raw 264.7 (RAW), and human astrocytes SVG-P12 were purchased from American Type Culture Collection (ATCC). Mouse colon cancer cell line MC38 was purchased from Kerafast. Mouse glioma cell line SB28 was provided by Hideho Okada of UCSF. Mouse GL261 glioma cell line was provided by Zhengping Zhuang of the National Institutes of Health (Bethesda, MD). The human SF268 glioma cell line was provided by Kunliang Guan of UCSD (San Diego, CA). All cell lines were regularly examined for mycoplasma contamination. SB28, RAW, SF268, SVG-P12, and L929 cells were maintained in DMEM with 10% FBS. THP1, MC38, and GL261 cells were maintained in RPMI 1640 with 10% FBS. THP1 macrophages were induced with PMA (100 nM) for 24 hours and rested for 24 hours with fresh medium before experimentation, unless otherwise specified. All cells were maintained at 37°C under 5% CO_2_.

### shRNA-mediated gene knockdown in cell lines

Lentiviral particles were produced by transfection of PLKO.1 shRNA lentiviral plasmid with psPAX2 (Addgene, 12260) and pMD2.G (Addgene, 12259) at 3:2:1 ratio into HEK293T cells to generate viral particles. shYAP, shTAZ, and shTEAD1/2/3 plasmids were previously described (44). Production of lentiviral particles and transduction method was performed similarly to what has been described here.

### STRN4 and PP2Ac overexpression

STRN4 (BC080283) and PP2Ac (BC054458) mouse cDNA clones were purchased from Transomic and cloned to AbVec vector from NovoPro. RAW cells were transfected by lipofectamine. After 48 hours, cells were collected for experiments.

### YAP2SA and YAP94SA overexpression

YAP2SA (S127A, S381A) and the YAP4SA mutant in pQCXIH retroviral plasmids were provided by Kunliang Guan. pQCXIH plasmids were transfected to HEK293 cells with pCMV-VSV-G and pCMV-GP plasmids at a ratio of 3:2:1. Retrovirus were collected after 48 hours. THP1 cells were transduced by spinoculation method as described above.

### In vitro experiments

#### BMDM.

Bone marrow was obtained from the hind legs of mice. Erythrocytes were lysed with Red Blood Cell Lysis Buffer (Sigma-Aldrich). BMDMs were generated from bone marrow cells with M-CSF (20 ng/mL) or 30% conditioned medium collected from L929 fibroblast cells. Culture medium was half changed on day 4. On day 7, macrophages were collected for further experimentation in DMEM with 10% FBS.

#### Human PBMC–derived macrophages.

Human PBMC was obtained from ProMab Biotechnologies Inc. PBMCs were then treated with M-CSF at 50 ng/mL. On day 6, macrophages were used for further experimentation in RPMI with 10% FBS.

### Surface staining and FACS

Cells were trypsinized and washed with FACS buffer (PBS, 2%FBS, 1 mM EDTA). Surface staining was performed by adding the surface antibodies to the cell suspension in 100 μl FACS buffer (Please see Supplemental Methods for details of reagents used.). After incubating for 30 minutes, cells were washed with FACS buffer and analyzed using the Cytek Aurora cytometer and analyzed using SpectroFlo (Cytek Bioscience) and FlowJo software.

#### Real time PCR.

Total RNA was extracted using PureLink RNA Mini Kit (Invitrogen) according to the manufacturer’s instructions. cDNA synthesis was performed with 0.5–1 μg of total RNA using High-Capacity cDNA Reverse Transcription Kit (Invitrogen). mRNA levels were measured with gene-specific primers using the SYBR Green PCR Master Mix (BioRad). The results were normalized to GAPDH or OAZ in human or mouse samples, respectively. The primers are shown in the attached Excel files.

#### Immune blotting and cell surface protein detection.

For immunoblot analysis, whole-cell lysates were prepared in RIPA lysis buffer (Thermo Fisher Scientific) containing Halt Protease and Phosphatase Inhibitor Cocktail (Thermo Fisher Scientific). The protein concentrations were determined by BCA Protein Assay Kits (Pierce). Protein samples between 20–30 μg were mixed with 4 × Laemmli buffer (BioRad) and denatured at 95°C for 10 minutes. Sample was separated by SDS-PAGE and transferred to nitrocellulose membranes (BioRad). Membranes were blocked with 3.5% BSA (Thermo Fisher Scientific) and incubated with primary antibodies overnight at 4°C followed by HRP-conjugated secondary antibodies for 2 hours at room temperature. Signal was detected using the ChemiDoc Imaging System (BioRad). Please see Supplemental Methods for a list of antibodies. Quantification, when performed, was done using BioRad Image Lab Software and labeled within the blot image.

#### Co-IP analysis.

For immunoprecipitation, cells were lysed with a mild lysis buffer (50 mM Tris at pH 7.5, 150 mM NaCl, 0.5% TritonX-100, 1 mM PMSF, and protease/phosphatase inhibitor cocktail) and centrifuged at 16,000*g* for 20 minutes at 4°C. Then the supernatants were incubated with the appropriate antibodies with rotation overnight at 4°C, and 15 μl Pierce Protein A/G Magnetic Beads (Thermo Fisher Scientific) were added for an additional 1.5 hour incubation. The immunoprecipitates were washed 3 times with mild lysis buffer, then the immunoprecipitated proteins were denatured by the addition of Laemmli Sample Buffer and boiling for 5 minutes, resolved by 9% or gradient (4–20%) SDS-PAGE and analyzed via Western blot.

#### RNA interference of PP2A regulatory subunit screening.

siRNA libraries against all known murine regulatory and scaffold subunits were purchased from Horizon Discovery. siRNA sequence information is included in the Supplemental Methods. siRNA transfection followed the manufacturer’s protocol (https://horizondiscovery.com/-/media/Files/Horizon/resources/Protocols/accell-delivery-protocol.pdf). Total RNA was extracted and expression of CXCL10 was quantified through reverse transcription PCR as described above.

### In vivo experiments

#### Animals.

Mice of both sexes, between the ages of 6 and 10 weeks were used for the study. *LysM^cre^PP2Ac^fl/fl^* on *C57BL/6* were provided by Bethany Moore at University of Michigan (Ann Arbor, Michigan, USA) as previously reported. WT *C57BL/6* mice were obtained from The Jackson Laboratory. All mice are maintained under pathogen-free conditions.

For s.c. tumor models, MC38 tumor cells (1 × 10^6^), B16F10 tumor cells (1 × 10^5^), and SB28 (1 × 10^5^) were s.c. injected on the right flank of *C57BL/6* mice in a 1-to-1 mix of PBS with Matrigel (Corning). An equal number of male and female mice were used. Tumor diameters were measured using calipers and volume was calculated. For radiotherapy, 7 days after implantation, tumors were irradiated with an X-ray radiator (MultiRad 350; Precision) with 3Gy daily for 3 consecutive days (3 × 3Gy). A lead shield was applied except over the right flank to achieve local radiation delivery to the tumor. For anti-PD-1 blockade, mice were treated with anti-PD-1 and IgG1 isotype antibodies i.p. at a dose of 200 μg per mouse on day 7 after tumor cell inoculation, then every 3 to 4 days for the duration of the experiment. For cGAMP treatment, 3μg of 2′3′-cGAMP in 50 μL of PBS was injected into the tumor at days 4, 8, and 11 after implantation. 50 μL of PBS was injected intratumorally in the control group. For CD8 depletion, mice were treated with IgG isotype or anti-CD8 depleting antibodies i.p. prior to tumor implantation (days –3, –2, and –1) followed by treatment twice weekly throughout the study. For IFN depletion, mice were treated with IgG1 isotype or IFNAR-blocking antibody i.p. on days 0 and 2 after tumor implantation followed by biweekly injections

For orthotopic brain tumor models, 8-to-10-week-old *C57BL/6* mice (male and female in equal numbers) were used for i.c. studies. Cell lines (GL261, SB28) were suspended in DMEM for inoculation. Mice were anesthetized with isoflurane, and 30,000 tumor cells were injected orthotopically in 3 μL. Using a stereotactic frame, a burr hole was formed on the skull via a 0.7 mm drill bit 1.5mm laterally to the right and 1.5mm rostrally from the bregma, and a noncoring needle (26s gauge; Hamilton) was used to inject the cells at a depth of 3mm into the brain from the burr hole. The skin incision was sutured. Mice were then monitored daily. Survival endpoint was defined as weight loss greater than 20% relative to baseline, a body condition score of less than 2,or presence of focal neurological deficits. For radiotherapy, 5 days after implantation, the mouse’s head was focally irradiated with an X-ray radiator (MultiRad 350; Precision), with 3Gy daily for 3 consecutive days (3 × 3Gy). A lead shield was applied to cover the mice except the cranium to achieve local radiation to the brain.

Please see supplemental information for further detailed methods.

#### Data availability.

RNA-Seq and scRNA-Seq data are available on GEO (GSE199271): https://www.ncbi.nlm.nih.gov/geo/query/acc.cgi?acc=GSE199271

### Statistics

No statistical methods were used to predetermine sample size. For cell-based experiments, biological triplicates were performed in each single experiment unless otherwise stated. Animal experiments were performed in *C57BL/6* mice. Animals were randomized into different groups after tumor cell inoculation; at least 9–10 mice were used for each group, unless otherwise indicated. Animals that failed to develop tumors were excluded from the analysis. Survival functions were estimated by the Kaplan-Meier methods and compared using the log-rank test. 2 tailed *t* tests and Mann-Whitney U tests were used to compare treatments versus control groups. One-way ANOVA models with and without Tukey’s multiple comparison test were used to compare continuous outcomes across multiple experimental groups, unless otherwise indicated in each figure legend. Bonferroni’s correction was used to adjust *P* values where appropriate. Mantel-Cox log-rank tests were used for survival analysis in some cases. Statistical analysis was performed using GraphPad Prism8 software (GraphPad Software, Inc.).

### Study approval

All animal work was approved by the IACUC at the University of Texas at Austin and UCSF.

## Author contributions

WSH and ROL conceived the project and designed the experiments. IM and OD performed most of the experiments, with help from all authors. BX performed all bioinformatic analysis. FT, ZW, and CYJW assisted with confocal experiments and analysis. EM contributed to in vitro and in vivo experiments. PC and RS assisted with cell culturing and Western blots. XC performed immunoprecipitation experiments. ZM provided experimental materials and scientific input. WM and ML provided scientific input and discussion. The order of co–first authors was based on the order with which the individuals joined the project.

## Supplementary Material

Supplemental data

Supplemental table 1

Supplemental table 2

## Version 1 02/09/2023

In-Press Preview

## Version 2 03/15/2023

Electronic publication

## Version 3 05/03/2023

Added author
