# Supplementary material for: PP2Ac/STRN4 negatively regulates STING-type I IFN signaling in tumor-associated macrophages
Source: J Clin Invest. 2023 Mar 15;133(6):e162139. doi: 10.1172/JCI162139 (PMC10014107; doi:10.1172/JCI162139)
Supplement: Supplemental data [file jci-133-162139-s009.pdf]

Supplementary Methods

CRISPR-mediated gene deletion in cell lines

CRISPR genomic editing technology was used to generate knock-out of genes in THP-1 and RAW cells. Cells were transfected with lentivirus encoding the guide RNA sequences cloned into the lentiCRISPRv2 plasmid (Addgene, 52961). To generate the viral particles, lentiCRISPRv2 with sgRNA plasmids were transfected together with psPAX2 (Addgene, 12260) and pMD2.G (Addgene, 12259) at ratio 3:2:1 into HEK293A cells. Medium was replaced with fresh DMEM after 24 hours then 48 hours later viral supernatant was collected. For THP-1, cells were transfected with the viral supernatant by spinoculation (900g for 30 mins) with polybrene (Sigma-Aldrich, 8 µg/ml). For RAW, cells were transfected with viral supernatant overnight with polybrene (8 µg/ml). 48 hours after transfection, cells were placed in 4 µg/ml puromycin for 2–3 d to select for transfected cells. Knockout efficiency was assessed using western blotting. To generate cGAS KO clones of SB28 and B16 cells, the sgRNA sequences were cloned into the pSpCas9(BB)-2A-Puro (PX459) plasmid (Addgene, 62988). Cells were then transfected with PX459-sgRNA plasmids. After 24-48h, 4 ug/ml puromycin were added for 24h-48h. Cells were then serially diluted and seeded into 96-well plates. After 2–3 weeks, several single-cell colonies were selected and expanded. Each clone was then validated for knockout efficiency using western blotting. One representative clone was selected for downstream application. All transfections were conducted using Lipofectamine 3000 (Thermo Fisher Scientific) at a ratio of 2.5 µg plasmid: 3.75 µL transfection reagent. The transfection dosage was determined by titration.

Immunofluorescence staining

For YAP visualization in THP1-macrophage *in vitro*. THP-1 cells were mounted on glass chamber slides (Millipore) and treated with PMA (100nM) for 24 hours (day 0). On day1 PMA was washed and replaced with tumor or astrocytes conditioned medium. On day 5, immunohistochemistry was performed. After washing twice with PBS, cells were fixed with 4% ice-cold PFA for 15 min at room temperature and washed three times with PBS for 5 min each afterwards. The cells were next permeabilized with 0.3% Triton X-100 in PBS for 15 min at room temperature and rinsed twice with PBS. Antigens were treated

with a blocking buffer consisting of 5% normal goat serum and 0.3% Triton in PBS for one hour at room temperature. Primary antibodies were then added at 1:200 dilutions of mouse anti-human YAP/TAZ antibodies (Santa Cruz, 101199) and incubated at 4 °C overnight. The cells were then washed and incubated with 1:200 dilutions of AF4888-labeled secondary goat anti-mouse antibodies (Abcam 150113) or Alex594-labelled secondary goat anti-rabbit antibodies (Abcam 150120), and then mounted onto glass slides using vectaSHIELD HardSet, Antifade mounting medium with DAPI. (Vector Laboratories H-1500). Confocal fluorescence images were collected using a ×40 oil-immersion objective (Nikon A1R confocal).

Quantification of Immunofluorescence staining

For quantification of YAP signaling from immunofluorescence imaging (in Figure 7B and Supplemental Figure 16A), the mean pixel intensity of the YAP channel was collected using the measure tool in Fiji (ImageJ). Acquisition parameters, such as lens objective, pinhole settings, and laser power, were kept consent. Five areas of 1024 X 1024 $pixels^2$ were randomly selected and imaged per sample. To adjust the background signal, the LUTs were changed equally among all images, then the composite images were split based on the color channel and the threshold was adjusted on the YAP staining channel to set the analysis region of interest (ROI). The total YAP intensity of each ROI was measured and normalized by the total number of pixels, adjusting for the pixel count variation within the region of interest among each of the five images. The mean pixel intensities were then averaged for each of the five regions.

***In vivo* immune cell profiling**

For intratumoural T cells and T-cell effector cytokine expression, single-cell suspensions were prepared from fresh tumor tissues using the Tumor Dissociation Kit (Miltenyi, 130-096-730) with the gentleMACS Octo Dissociator (Miltenyi) per manufacture instruction. For draining lymph nodes, tissues were dissociated in a 70 µm mesh and washed several times with FACS buffer to obtain single cell suspension. Fc receptors were blocked with TruStain FcX anti-mouse CD16/32 (BioLegend,

101319) in 20 μg/mL for 10 mins at 4°C. Cells were stained with surface markers in 4°C for 30 mins. For cytokine staining, cells were incubated with Cell Stimulation Cocktail (Thermo Scientific, 00-4975) in RPMI medium containing PMA ionomycin, brefeldin A and monensin at 37 °C for 4 h before undergoing surface staining. The cells were then washed and resuspended in IC Fixation Buffer (Thermo Scientific, 00-8222) at room temperature for 20 mins then wash with Perm/Wash buffer (Thermo Scientific, 00-8333). Cells were then stained with specific intracellular markers. All the samples were read using the Cytek Aurora cytometer and analyzed using SpectroFlo (Cytek Bioscience) and FlowJo software.

Survival analysis

Normalized expression levels for GBM, LGG, SKCM and BRCA were downloaded from Broad GDAC Firehose portal (level 3 RSEM) (Broad). For each patient data, mean expression levels of the marker genes of the cluster of interest were calculated. Patients were then grouped into 4 quartiles that Q1 were expressed lower level of marker genes. Then survival analysis was performed at cBioportal in Merged Cohort of LGG and GBM(52), SKCM and BRCA(53, 54).

RNA sequencing of BMDM

BMDM from WT and PP2Ac were generated as described above. They were them treated with STING agonist DMAXX (InvivoGen), 10 ug/ml, for 4h before RNA extraction. Total RNA was isolated using the PureLink™ RNA Mini Kit (Invitrogen, 12183025). RNA sequencing was performed by the University of Texas Genomic Sequencing and Analysis Facility. Poly(A) enrichement was performed. mRNA was isolated from total RNA using the Poly(A) Purist-MAG kit (ThermoFisher). Briefly, total RNA was combined in a 96-well plate with washed and resuspended Oligo (dT) MagBeads. The mixture was heated in a thermocycler set to 70°C for 5 minutes, followed by incubation at room temperature with gentle vortexing for 30 minutes. Subsequently, the beads were captured and washed, and bound poly(A) RNA was eluted in water. mRNA quality was assessed on the Agilent Bioanalyzer using the Agilent RNA 6000 Pico kit (Agilent). Libraries were prepared at the University of Texas Genomic

Sequencing and Analysis Facility according to manufacturer's instructions for the NEBNext Ultra II Directional RNA kit (NEB, product number E7760). The resulting libraries tagged with unique dual indices were checked for size and quality using the Agilent High Sensitivity DNA Kit (Agilent). Library concentrations were measured using the KAPA SYBR Fast qPCR kit and loaded for sequencing on the NovaSeq 6000 instrument (paired end 2X150). Reads were first trimmed for adapters using fastp (v0.20.0)(52), then counted in two ways at gene exons 3'UTRs either by pseudoalignment approach kallisto (v0.46.1, parameters "--single -l 200 -s 20") or alignment to the mouse genome mm10(Gencode GRCm38) by STAR (version 2.7.1a, parameters: "--outFilterMultimapNmax 20 --alignSJoverhangMin 8 --alignSJstitchMismatchNmax 5 -1 5 5 --alignSJDBoverhangMin 10 --outFilterMismatchNmax 999 --outFilterMismatchNoverReadLmax 0.04 --alignIntronMin 20 --alignIntronMax 100000 --alignMatesGapMax 100000 --outSAMmapqUnique 60 --outSAMmultNmax 1 --outSAMstrandField intronMotif --outSAMattributes NH HI AS nM NM MD --outSAMunmapped Within --chimSegmentMin 12 --chimJunctionOverhangMin 12 --chimSegmentReadGapMax 3 --chimMultimapNmax 10 --chimMultimapScoreRange 10 --chimNonchimScoreDropMin 10 --chimOutJunctionFormat 1 --quantMode TranscriptomeSAM GeneCounts --twopassMode Basic --peOverlapNbasesMin 12 --peOverlapMMp 0.1")(53) and counted by HTSeq(v0.11.2)(54) based on GENCODE mouse gene annotation (vM22)(55). STAR results were chosen for following analysis since they got more reads counts that all samples achieved 1.5M reads counts. After normalization by trimmed mean of M-values normalization (edgeR v3.34.0)(56), Voom (limma v3.48.3)(57)was used to identify differential expressed genes. Pathway enrichment analysis were then performed at Enrichr server(58) on significant differential expressed genes. GSEA(v4.0.3)(59). Prerank mode were ran with MSigDB gene sets database (v7.1)(60) against the gene rank by log2 fold change. YAP targeted genes were extracted from Supplementary table of Zhao et al.(46). For human and murine glioma analysis, raw counts were downloaded from Brain Time portal(45, 47) and differential analysis were performed using Voom like mentioned above.

Mouse scRNASeq analysis

SB28 tumors were inoculated in the flank, or the brain, as described above, in $PP2Ac^{WT}$ or $PP2Ac^{KO}$ mice. 18 days after implantation, tumor tissue was harvested, and single cell suspensions were obtained using Tumor Dissociation Kit (Miltenyi, 130-096-730) and the gentleMACS Octo Dissociator (Miltenyi) per manufacture instruction. Single cell suspensions were processed in the University of Texas Genomic Sequencing and Analysis Facility. Cell suspensions were loaded on the Chromium Controller (10X Genomics) and processed for cDNA library generation following the manufacturer's instructions for the Chromium NextGEM Single Cell 3' Reagent Kit v3.1 (10X Genomics). The resulting libraries were examined for size and quality using the Bioanalyzer High Sensitivity DNA Kit (Agilent) and their concentrations were measured using the KAPA SYBR Fast qPCR kit (Roche). Samples were sequenced on the NovaSeq 6000 instrument (paired end, read 1: 28 cycles, read 2: 90 cycles) with a targeted depth of 40,000 reads/cell. Read processing, read alignment, cell-barcode demultiplexing, and gene-specific UMI counting was performed with CellRanger (v3.0.2)(61) using the mouse references mm10(ensemble version r97, MH029771.1-sfGFP and GQ357182.1-eGFP CDS sequences were downloaded from Genbank and added to the references, see DOI:10.6084/m9.figshare.19342418 for details). Distinct samples were processed independently and reviewed for quality that each sample achieved ~8000 cells, > 1200 expressed genes, > 90% reads in cells. All 4 samples were then aggregated by CellRanger for downstream analyses. Seurat (version 4.0.6)(62) were then used for analysis based on filtered feature barcode matrices. Briefly, cells with < 200 expressed genes or genes expressed in < 3 cells were filtered out from analysis. SCTransform (Seurat function) were used to normalize the data using top 3000 variable genes and regressing out the effect of mitochondrial expression percentage and cell cycle scores(63). We then performed Principal Component Analysis (PCA) on the scaled genes and selecting the top 10 PCs for following analysis because the standard deviation plateaued after top 10 PCs in ElbowPlot (Seurat function). tSNE and UMAP have been ran and clusters have been identified using default parameters from Seurat. Cd45+eGFP- cell/clusters (Clusters 6, 10-15, 17, 21-23 or Ptprc

> 0, exclude eGFP > 0) were then selected for downstream analysis separately by tissues (Details command lines used, QC files, UMAP/tSNE projection and clustering meta data could be found at DOI:10.6084/m9.figshare.19342418). For each analysis result, FindMarker function (Seurat) were used to identify markers ("lr" mode, min.pct = 0.20, min.diff.pct = 0.1, logfc.threshold = 0.25). Known markers (Supplementary table 1 of DOI:10.1038/s41467-021-21407-w), scMRMA(v1.0) and SCSA(v1.1) were used to facilitate cell type assignment(64, 65). GSEA(v4.0.3)(59) Prerank mode were ran with MSigDB(v7.1)(60) against the gene rank by log2 fold change.

Reagents

Antibodies

Anti-mouse PD-1 (RMP1-14)	BioXcell	BE0146
Ctrl IgG (Syrian hamster IgG control for anti-PD-1)	BioXcell	BE0087
Anti-mouse IFNAR-1 (MAR1-5A3)	BioXcell	BE0241
Ctrl IgG (Mouse IgG1 control for anti-IFNAR-1)	BioXcell	BE0083
Anti-mouse CD8a (2.43)	BioXcell	BE0061
Ctrl IgG (Rat IgG2b control for anti-CD8a)	BioXcell	BE0090
Rabbit monoclonal Anti- Phospho-IRF-3 (Ser396) (4D4G)	Cell Signaling Technology	Cat#4947
Rabbit monoclonal Anti-IRF-3 (D83B9)	Cell Signaling Technology	Cat#4302
Rabbit monoclonal Anti-Cyclophilin A (D2Y4M)	Cell Signaling Technology	Cat#51418
Rabbit monoclonal Anti- β-Actin (13E5)	Cell Signaling Technology	Cat#4970
Rabbit monoclonal Anti-Stat1 (D1K9Y)	Cell Signaling Technology	Cat#14994
Rabbit monoclonal Anti-Phospho-Stat1 (Tyr701) (58D6)	Cell Signaling Technology	Cat#9167
Rabbit monoclonal Anti-MOB1 (E1N9D)	Cell Signaling Technology	Cat#13730
Rabbit monoclonal Anti- Phospho-MOB1 (Thr35) (D2F10)	Cell Signaling Technology	Cat#8699

Rabbit polyclonal Anti-MST1	Cell Signaling Techonolgy	Cat#3682
Rabbit monoclonal Anti- Phospho-MST1 (Thr183)/MST2 (Thr180) (E7U1D)	Cell Signaling Technology	Cat#49332
Rabbit polyclonal Anti-YAP	Cell Signaling Technology	Cat#4912
Rabbit monoclonal Anti- NF-κB p65 (D14E12)	Cell Signaling Technology	Cat#8242
Rabbit monoclonal Anti- Phospho-NF-κB p65 (Ser536) (93H1)	Cell Signaling Technology	Cat#3033
Rabbit monoclonal Anti- Phospho-YAP (Ser127) (D9W2I)	Cell Signaling Technology	Cat#13008
Anti-Rabbit IgG HRP Linked Secondary	Cell Signaling Technology	Cat#7074
Anti-Mouse IgG HRP Linked Secondary	Cell Signaling Technology	Cat#7076
Mouse monoclonal Anti-PP2A-Cα/β (1D6)	Santa Cruz Biotechnology	Cat#80665
Mouse monoclonal Anti-YAP1 (63.7)	Santa Cruz Biotechnology	Cat#101199
Rabbit polyclonal Anti-MST1-2/STK3-4	Bethyl Laboratories	Cat# A300-466A
FITC anti-mouse CD80 (16-10A1)	BioLegend	Cat#104705
PE anti-mouse CD86 (GL-1)	BioLegend	Cat#105007
BV650 anti-mouse I-A/I-E (M5/114.15.2)	BioLegend	Cat#107641
APC/Cyanine7 anti-mouse CD8a (53-6.7)	BioLegend	Cat#100714
Brilliant Violet 510 anti-mouse CD45 (30-F11)	BioLegend	Cat#103137
APC anti-mouse IFN-γ (XMG1.2)	BioLegend	Cat#505810
PE anti-mouse CD3ε (KT3.1.1)	BioLegend	Cat#155608
Purified anti-mouse CD4 (GK1.5)	BioLegend	Cat#100402
Brilliant Violet 421 anti-mouse TNF-α (MP6-XT22)	BioLegend	Cat#506327
APC anti-mouse F4/80 (BM8)	BioLegend	Cat#123116

Brilliant Violet 421™ anti-mouse CD206 (C068C2)	BioLegend	Cat#141717

Chemicals, peptides, and recombinant proteins

2'3'-cGAMP	InvivoGen	tlrl-nacga23
DMXAA	InvivoGen	tlrl-dmx
M-CSF	R&D system	416-ML-010/CF
LB-100	Selleck Chemicals	S7537
XMU-MP-1	Selleck Chemicals	S8334
PMA	Millipore Sigma	16561-29-8
Recombinant Mouse IFN-gamma	R&D system	485-MI-100/CF

Experimental models: cell lines

Mouse cell line: B16-F10	ATCC	CRL-6475
Mouse cell line: MC38	Kerafast	ENH204-FP
Mouse cell line: GL261	Zhuang Lab (Bethesda, MD)	N/A
Mouse cell line: SB28	Okada Lab (San Francisco, CA)	N/A
Mouse cell line: RAW 264.7	ATCC	TIB-71
Mouse cell line: L-929	ATCC	CCL-1
Human cell line: THP1	ATCC	TIB-202
Human cell line: SF268	Guan Lab (San Diego, CA)	N/A
Human astroglia cell line: SVG P12	ATCC	CRL-8621

Experimental models: organisms/strains

Mouse: C57BL/6J	The Jackson Laboratory	JAX: 000664
Mouse: LysM^{cre}PP2Ac$^{fl/fl}$	Moore Lab (Ann Arbor, MI)	N/A

Critical commercial assays

LegendPlex Mouse Anti-Virus Response Panel	BioLegend	Cat#740621

Oligonucleotides

sgRNA targeting sequence: Mouse *cGAS#1:* CGAGGCGCGGAAAGTCGTAA (used for SB28)	Synthego	N/A

sgRNA targeting sequence: Mouse *cGAS#2:* TAGCTTGTCCGGTTCCTTCC (used for B16)	Synthego	N/A
sgRNA targeting sequence: Mouse *STRN4#1:* GGCTGAAGATTACCTTCATG	Synthego	N/A
sgRNA targeting sequence: Human *PP2Ac#1:* AACGCATCACCATTCTTCGA	Synthego	N/A
sgRNA targeting sequence: Human *STRN4#1:* AACGCATCACCATTCTTCGA	Synthego	N/A
shRNA targeting sequence: Human shYAP: 5' GCCACCAAGCTAGATAAAGAAC 3'	Meng Lab (Miami, FL)	N/A
shRNA targeting sequence: Human shTAZ: 5' GCGTTCTTGTGACAGATTATAC 3'	Meng Lab (Miami, FL)	N/A
shRNA targeting sequence: Human shTEAD1/3/4: 5' TGATCAACTTCATCCACAAGC3'	Meng Lab (Miami, FL)	N/A
Primer: human *IFNβ* forward: AGGACAGGATGAACTTTGAC	IDT	N/A
Primer: human *IFNβ* reverse: TGATAGACATTAGCCAGGAG	IDT	N/A
Primer: human *CXCL*10 forward: GTGGCATTCAAGGAGTACCTC	IDT	N/A
Primer: human *CXCL*10 reverse: TGATGGCCTTCGATTCTGGATT	IDT	N/A
Primer: human *CXCL*9 forward: CCAGTAGTGAGAAAGGGTCGC	IDT	N/A
Primer: human *CXCL*9 reverse: AGGGCTTGGGGCAAATTGTT	IDT	N/A
Primer: human *ISG*15 forward: GCGAACTCATCTTTGCCAGTA	IDT	N/A
Primer: human *ISG*15 reverse: CCAGCATCTTCACCGTCAG	IDT	N/A
Primer: human *GAPHD* forward: GAAGGTGAAGGTCGGAGTC	IDT	N/A
Primer: human *GAPHD* reverse: GAAGATGGTGATGGGATTTC	IDT	N/A
Primer: mouse *IFNβ* forward: AGCTCCAAGAAAGGACGAACA	IDT	N/A
Primer: mouse *IFNβ* reverse: GCCCTGTAGGTGAGGTTGAT	IDT	N/A
Primer: mouse *CXCL*10 forward: CCAAGTGCTGCCGTCATTTTC	IDT	N/A
Primer: mouse *CXCL*10 reverse: GGCTCGCAGGGATGATTTCAA	IDT	N/A
Primer: mouse *CXCL9* forward: GGAGTTCGAGGAACCCTAGTG	IDT	N/A
Primer: mouse *CXCL9* reverse: GGGATTTGTAGTGGATCGTGC	IDT	N/A
Primer: mouse ISG15 forward: GGTGTCCGTGACTAACTCCAT	IDT	N/A
Primer: mouse ISG15 reverse: TGGAAAGGGTAAGACCGTCCT	IDT	N/A
Primer: mouse OAZ forward: TTATTGCTGTTTAAGATGGTCAG	IDT	N/A
Primer: mouse OAZ reverse: GAACGAGATCACTTTATTGGATT	IDT	N/A

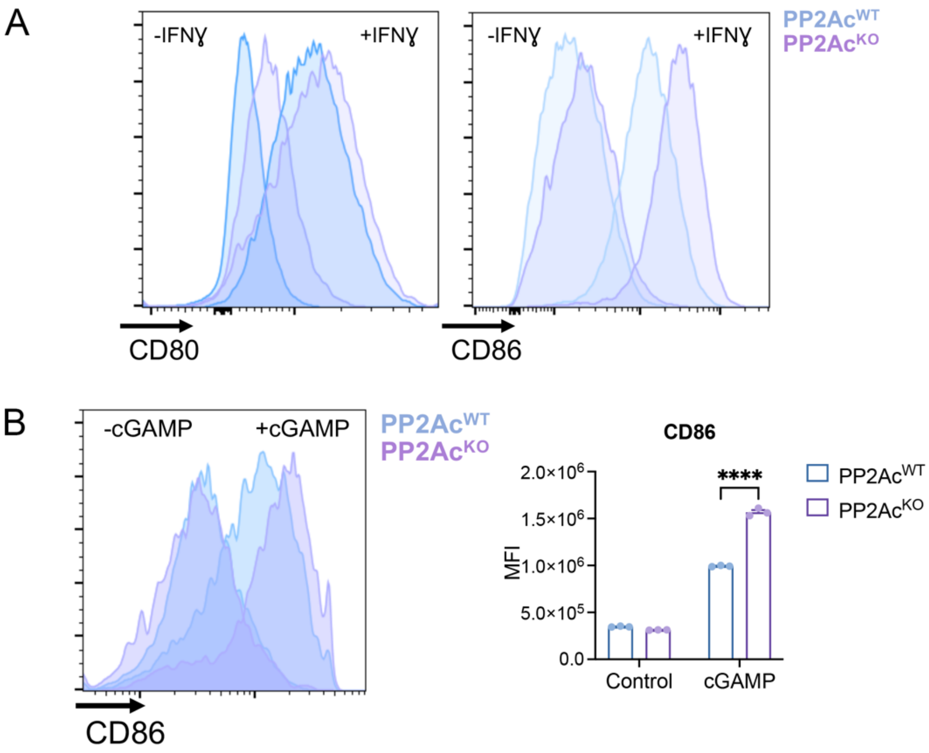

Supplementary Figure 1, related to Figure 1. PP2Ac^{KO} enhanced activation markers of BMDM in response to IFNg or cGAMP. (A) PP2Ac^{KO} and PP2Ac^{WT} BMDM were treated with IFNγ (10ng/ml) for 24 hours before flow cytometry analysis was performed for CD80, CD86 and MHCII expression. Representative FACs plot of CD80 and CD86 expression +/- IFNγ treatment in PP2Ac^{KO} and PP2Ac^{WT} BMDM. (B) PP2Ac^{KO} and PP2Ac^{WT} BMDM were treated with cGAMP (10ug/ml) for 24 hours before flow cytometry analysis was performed for CD86. Representative FACs plot +/- cGAMP treatment in PP2Ac^{KO} and PP2Ac^{WT} BMDM. Data are from one experiment representative of two independent experiments with similar results. Error bars depict SEM. P values were calculated by unpaired t-test (****P<0.0001).

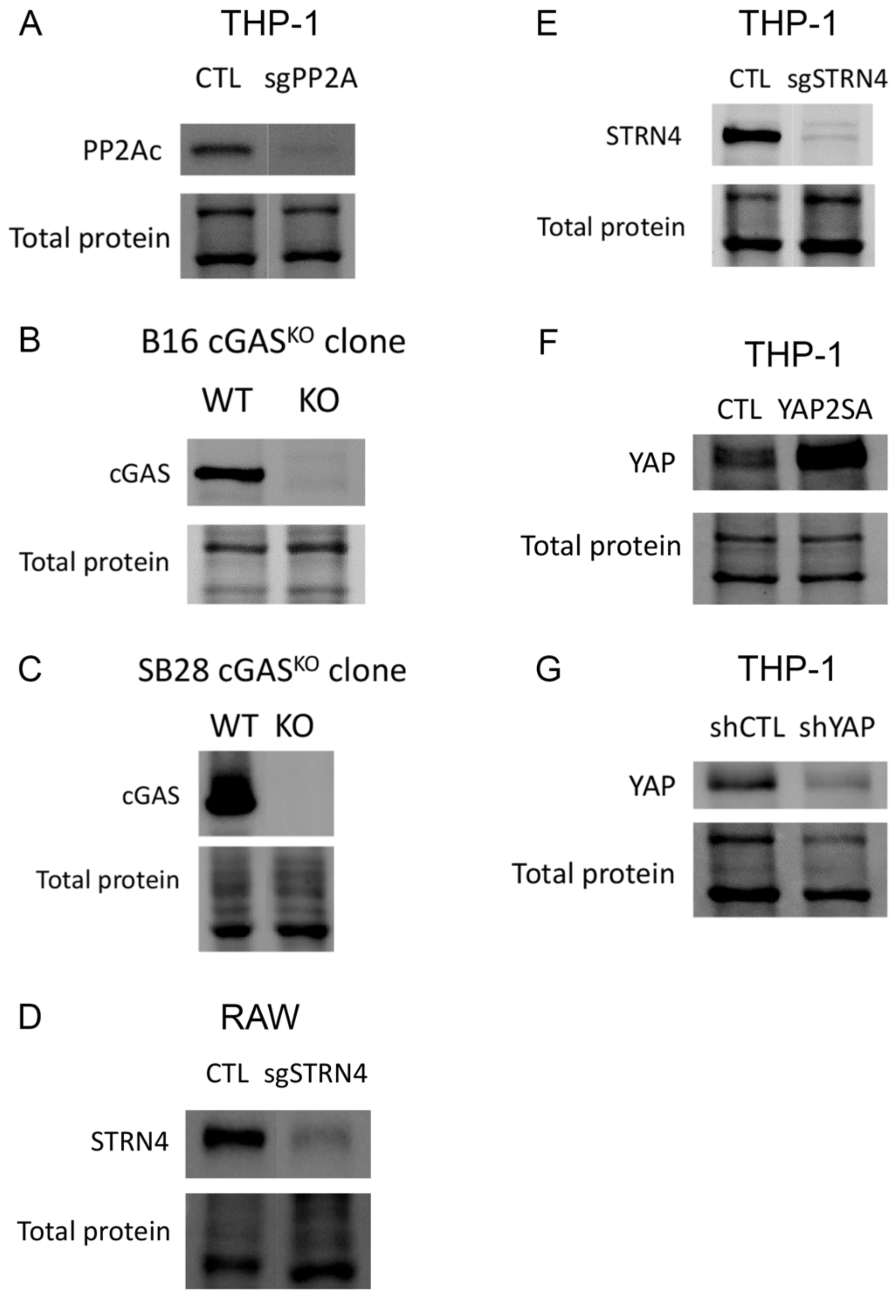

Supplementary Figure 2. Western blot confirmation of genetically modified cell lines used. (A) THP-1 PP2Ac^{KO}. (B) B16 cGAS^{KO}. (C) SB28 cGAS^{KO}. (D) RAW STRN4^{KO}. (E) THP-1 STRN4^{KO}. (F) THP-1 YAP2SA. (G) THP-1 shYAP KD.

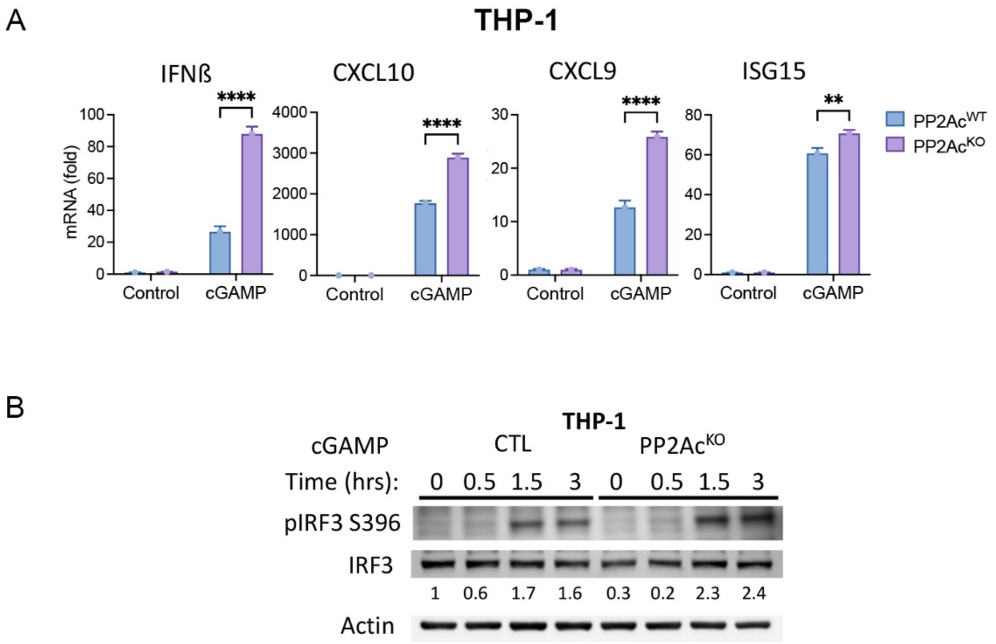

Supplementary Figure 3, related to Figure 1. PP2Ac^{KO} enhanced STING-Type I IFN signaling pathway in response to cGAMP in THP-1 derived macrophage. Human THP-1 cells were differentiated into macrophages by phorbol myristate acetate (PMA) for 24 hours (A) Cells were then treated with cGAMP stimulation (10ug/ml) and harvested 4 hours after. Expression of IFNβ and IFN response genes (CXCL10, CXCL9, ISG15) were measured via RT-PCR. (B) Protein expression of THP-1 differentiated macrophage was analyzed by immunoblotting at different time points after cGAMP (10ug/ml) treatment. Data are from one experiment representative of two independent experiments with similar results. Error bars depict SEM. P values were calculated by unpaired t-test (**P<0.01, ****P<0.0001).

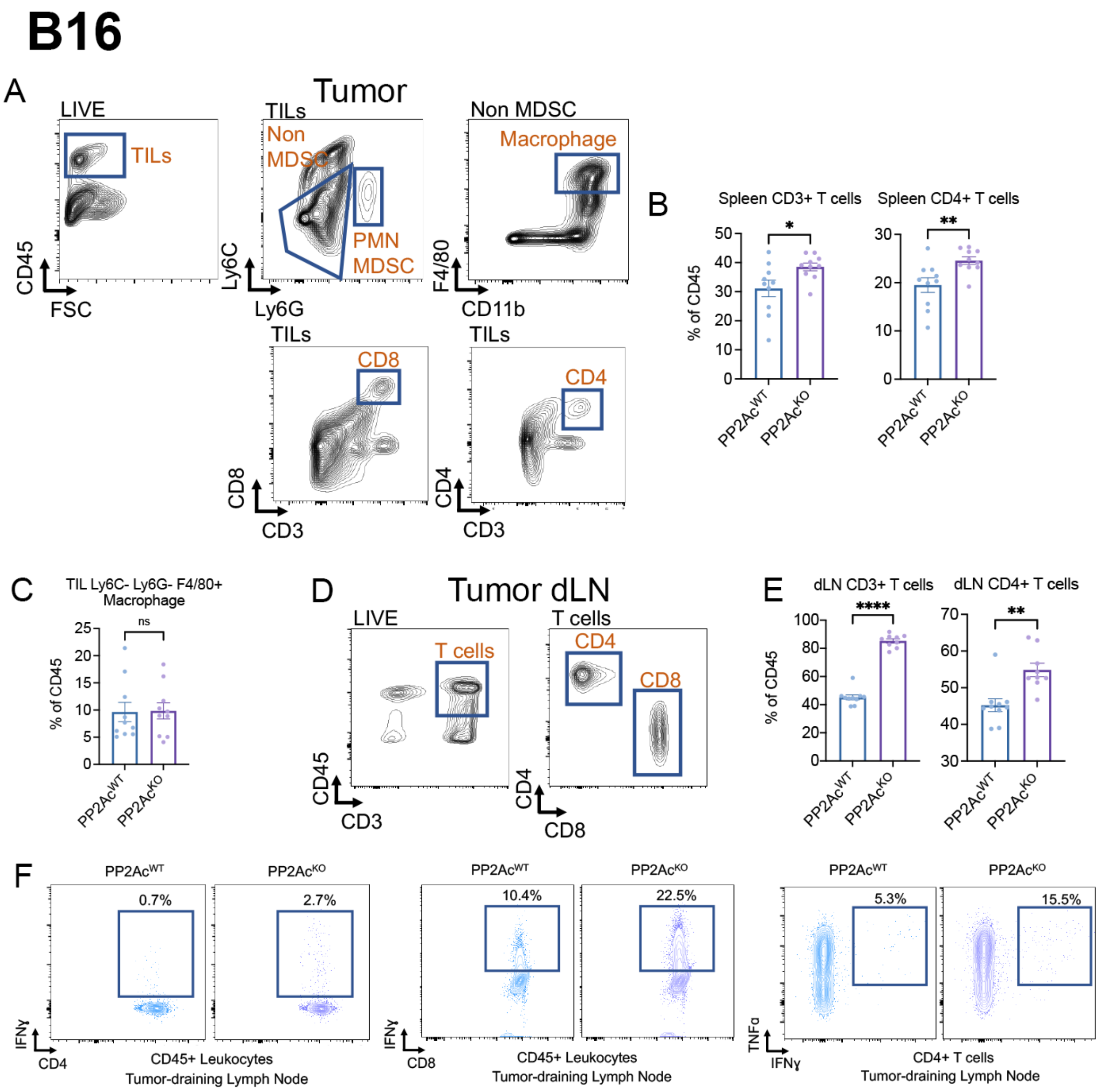

Supplementary Figure 4, related to Figure 2. Analysis of immune cells in B16 tumor bearing mice. B16 tumors were implanted subcutaneously in the right thoracic flank of *LysM^cre PP2Ac^fl/fl* or WT mice. Mice were euthanized on day 10. Tumor, tumor-draining lymph node (dLN) and spleen were harvested and analyzed by flow cytometry (n=9-10). (A) Gating strategy for analyzing tumor infiltrating leukocytes (TILs). (B) Quantification of $CD3^+$ and CD4+ T-cells in spleen as a % of $CD45^+$. (C) Quantification of $Ly6C^-Ly6G^-F480^+$ macrophage in TILs. (D) Gating strategy for T cells in dLN. (E) Quantification of $CD3^+$ and CD4+ T-cells in dLN as a % of CD45. (F) Representative FACS plots of dLN $CD4^+$ and $CD8^+$ T cells after ex-vivo stimulation with PMA/ionomycin in conjunction with protein transport inhibitor. P values were calculated by unpaired t-test (*P<0.05, **P<0.01, ****P<0.0001, ns P>0.05).

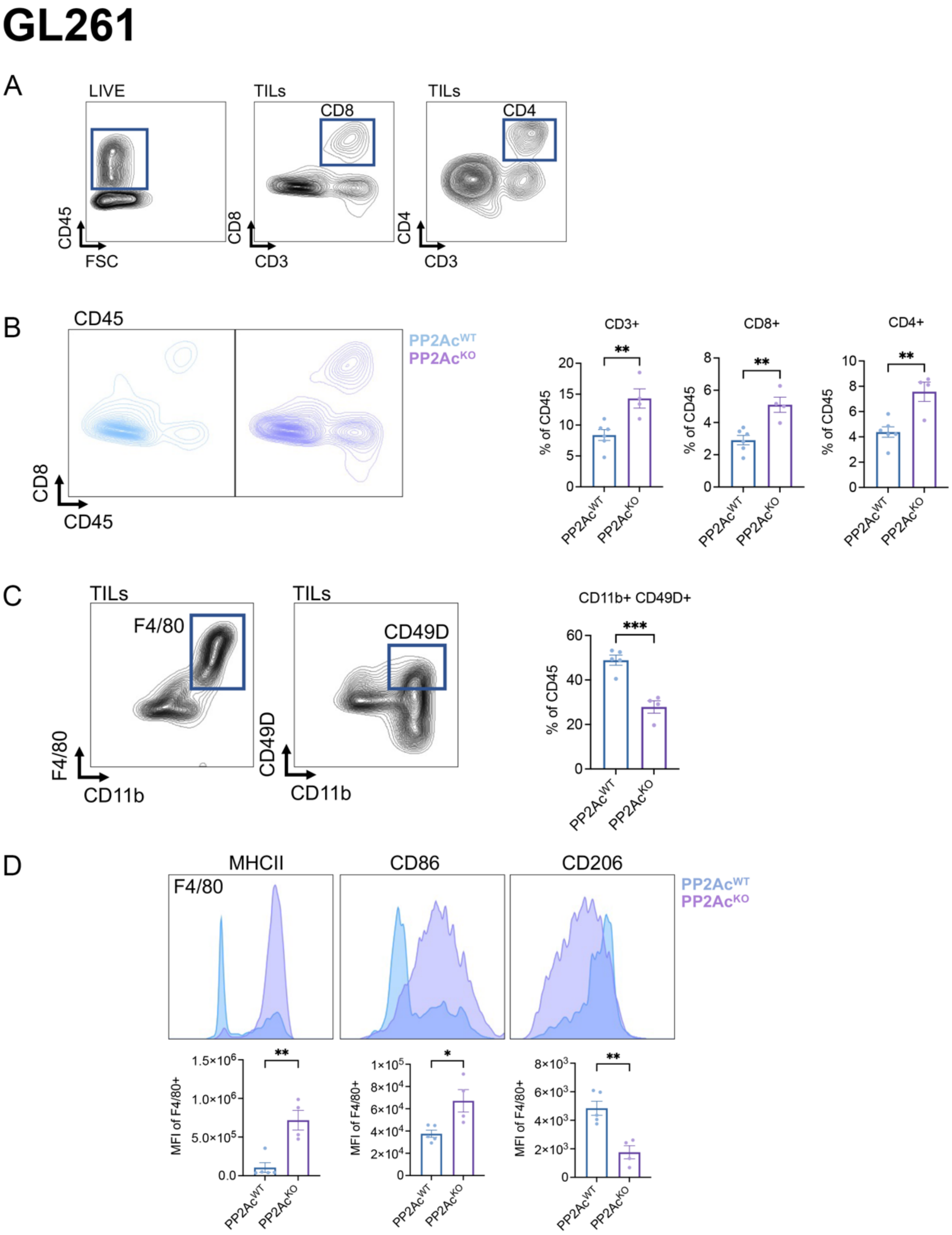

Supplementary Figure 5, related to Figure 2. Analysis of immune cells in GL261 orthotopic glioma bearing mice. GL261 tumors were implanted orthotopically in the brain of *LysM^{cre}PP2Ac$^{fl/fl}$* or WT mice. Mice were euthanized on day 12. Tumors were harvested and analyzed by flow cytometry (n=4-5). (A) Gating strategy for analyzing TILs. (B) Quantification of $CD4^+$,$CD8^+$ TILs. Representative FACS plots of $CD8^+$ TILs. (C) Gating strategy for analyzing $CD11b^+$,$F4/80^+$ macrophage or $CD11b^+$ $CD49D^+$ blood monocyte derived macrophage. Quantification of $CD11b^+$ $CD49D^+$ TILs as % of $CD45^+$. (D) Quantification of MHCII, CD86, CD206 expression in $CD11b^+$ $F4/80^+$ macrophages. Representative FACs plot of respective markers. Error bars depict SEM. P values were calculated by unpaired t-test (*P<0.05, **P<0.01, ***P<0.001, ****P<0.0001).

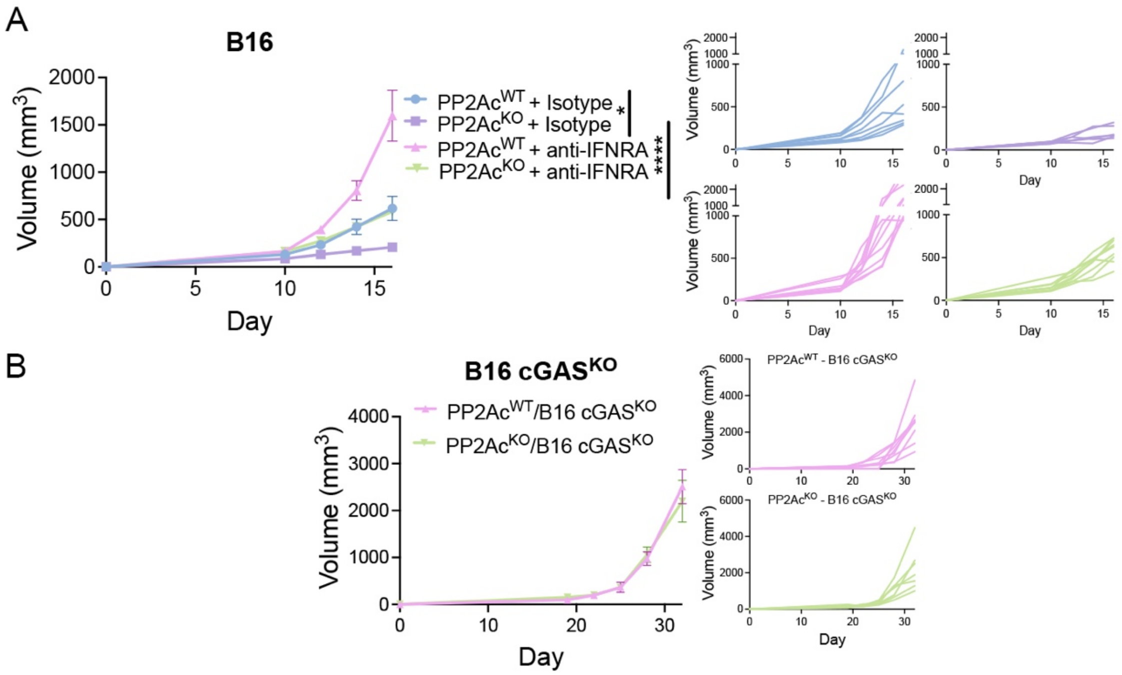

Supplementary Figure 6, related to Figure 3. Enhanced tumor control of macrophage PP2Ac deficiency depends on Type I IFN signaling and tumor derived cGAMP. (A) *LysMcrePP2Acfl/fl* or WT C57BL/6 mice were inoculated with 0.1×10^6 B16 cells (n=8). Mice were given 100 µg intra-tumoral injection of anti-IFNAR-1 (clone MAR1-5A3, BioXCell) or isotype control (mouse IgG1, BioXcell) on day 0, 2 then 2x/week until endpoint. Tumor growth curves with individual and mean tumor volume over time. (B) *LysMcrePP2Acfl/fl* or WT mice were inoculated with 0.1×10^6 B16 cGAS^{KO} (n=8) cells subcutaneously in the right thoracic flank. Tumor growth curves with individual and mean tumor volume over time. Error bars depict SEM. P values were calculated by one-way ANOVA with Tukey's multiple comparison test (*P<0.05, ****P<0.0001).

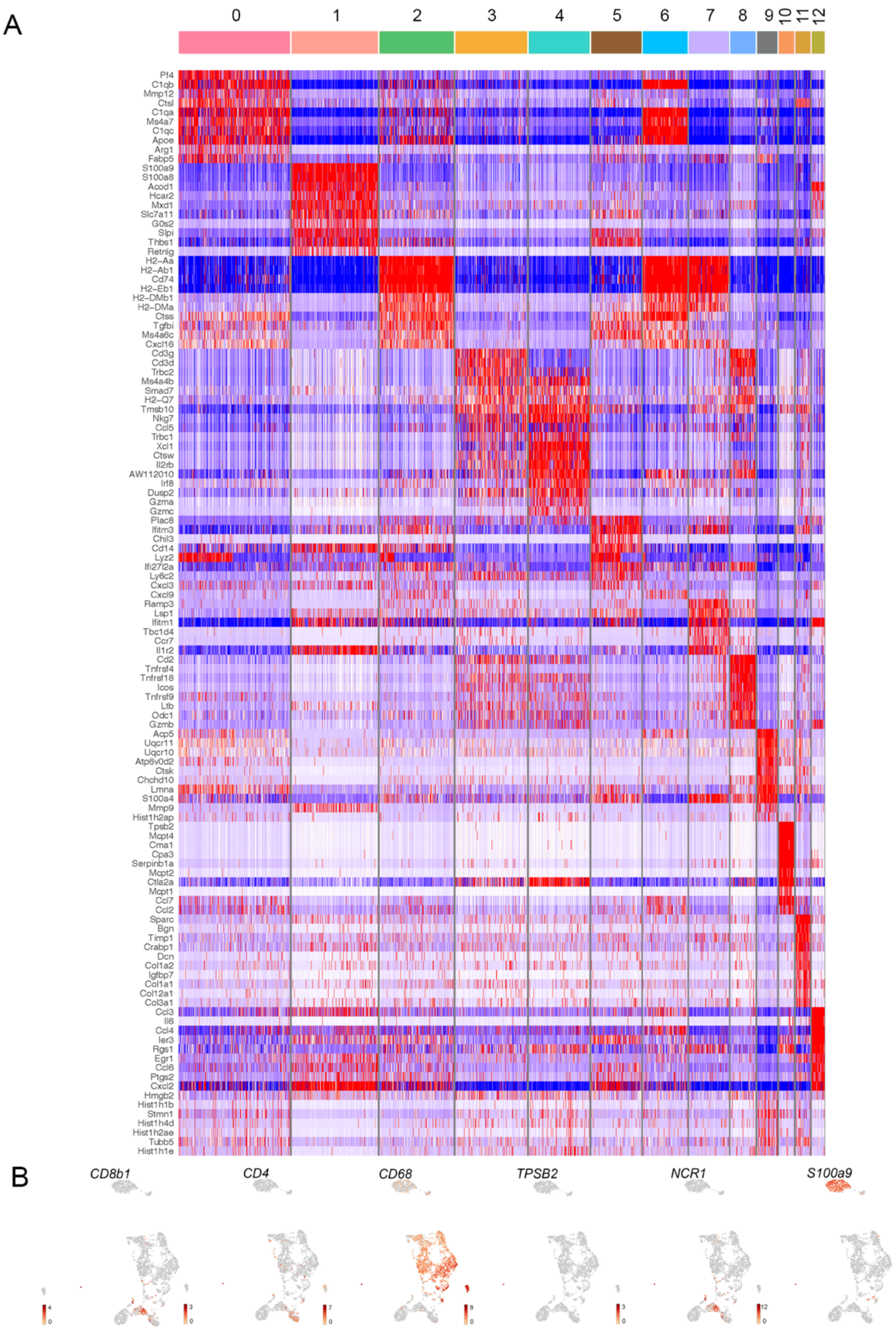

Supplementary Figure 7, related to Figure 4. scRNA-seq analysis reveals an altered immune landscape with myeloid specific PP2Ac deficiency in s.c. SB28 tumor. (A) Heatmap displaying normalized expression of the top 10 up-regulated genes in each Seurat cluster. (B) Canonical markers were used to identified major $CD45^{+}$ immune population.

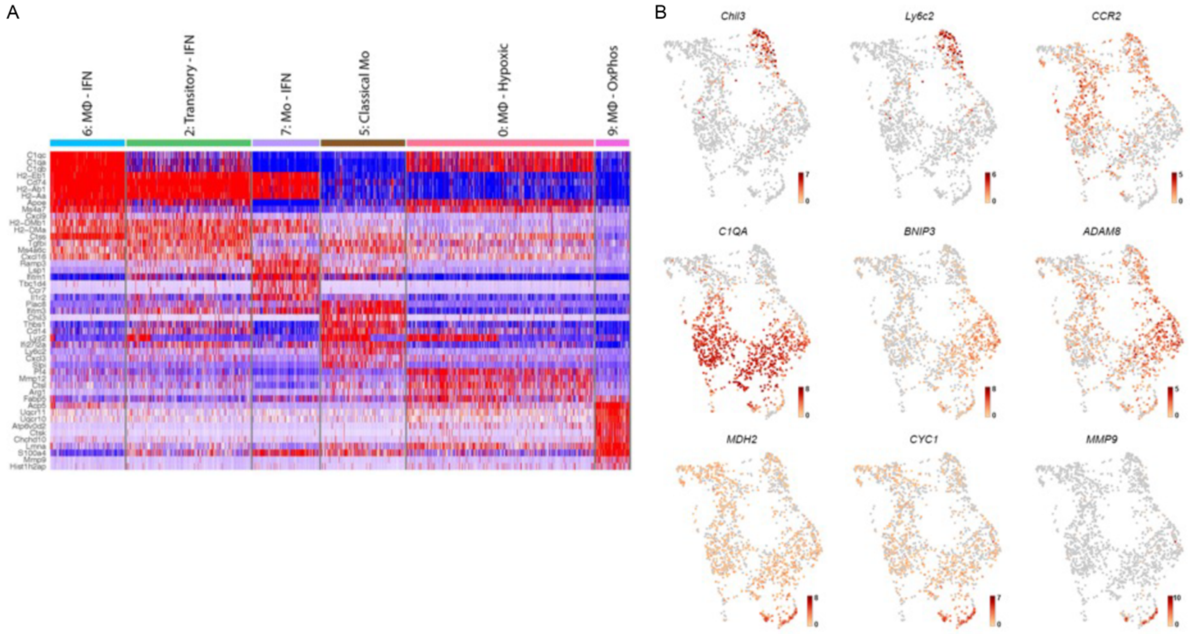

Supplementary Figure 8, related to Figure 4. scRNA-seq analysis of TAMS in s.c. SB28 tumor. (A) Heatmap displaying normalized expression of the top 10 up-regulated genes in each TAMs cluster. (B) UMAP showing key signature genes differentiating TAMs subsets.

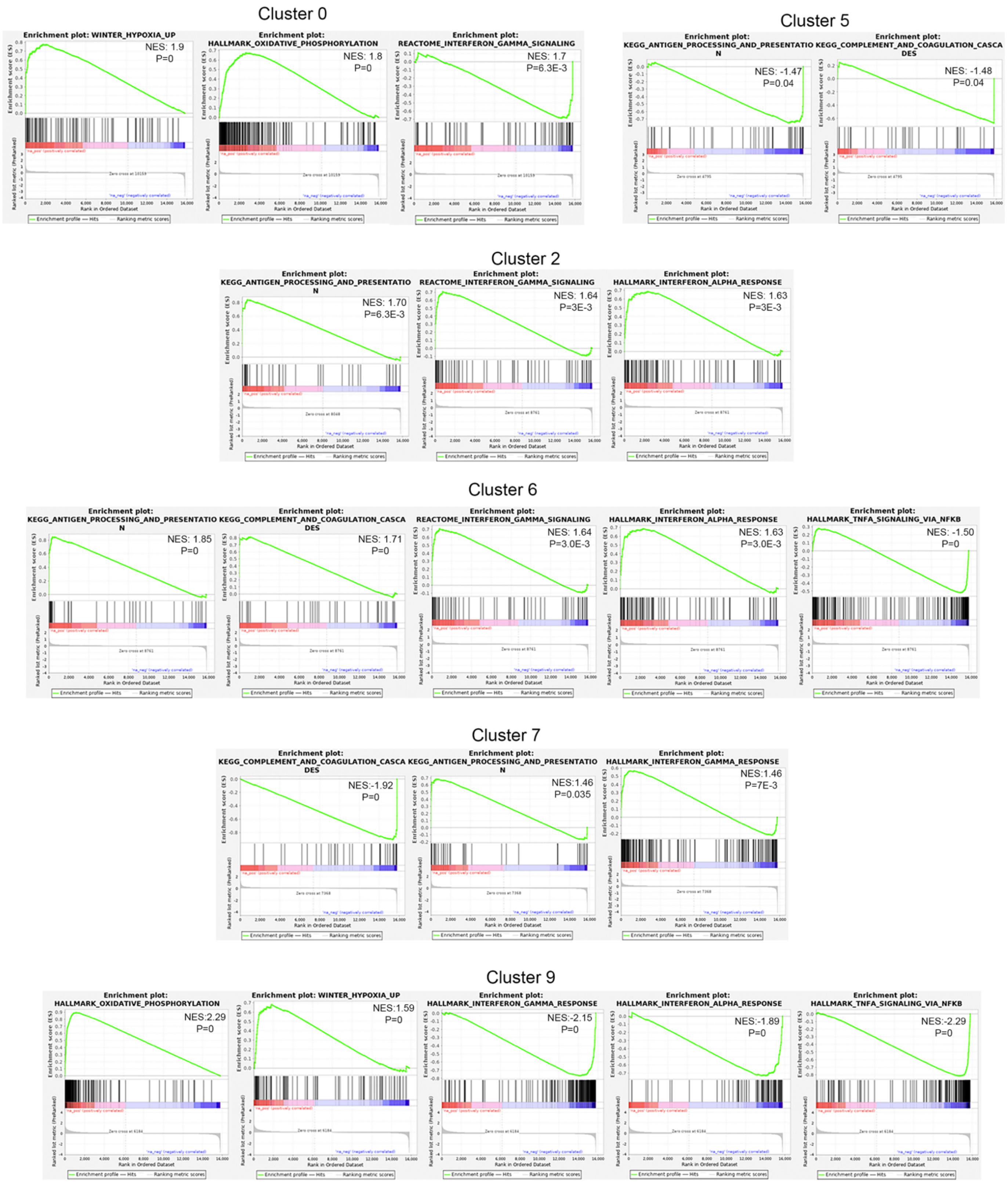

Supplementary Figure 9, related to Figure 4. Selected GSEA plots of significant gene signature pathway upregulated or downregulated in each TAMs cluster in scRNA-seq analysis of s.c. SB28 tumor.

A

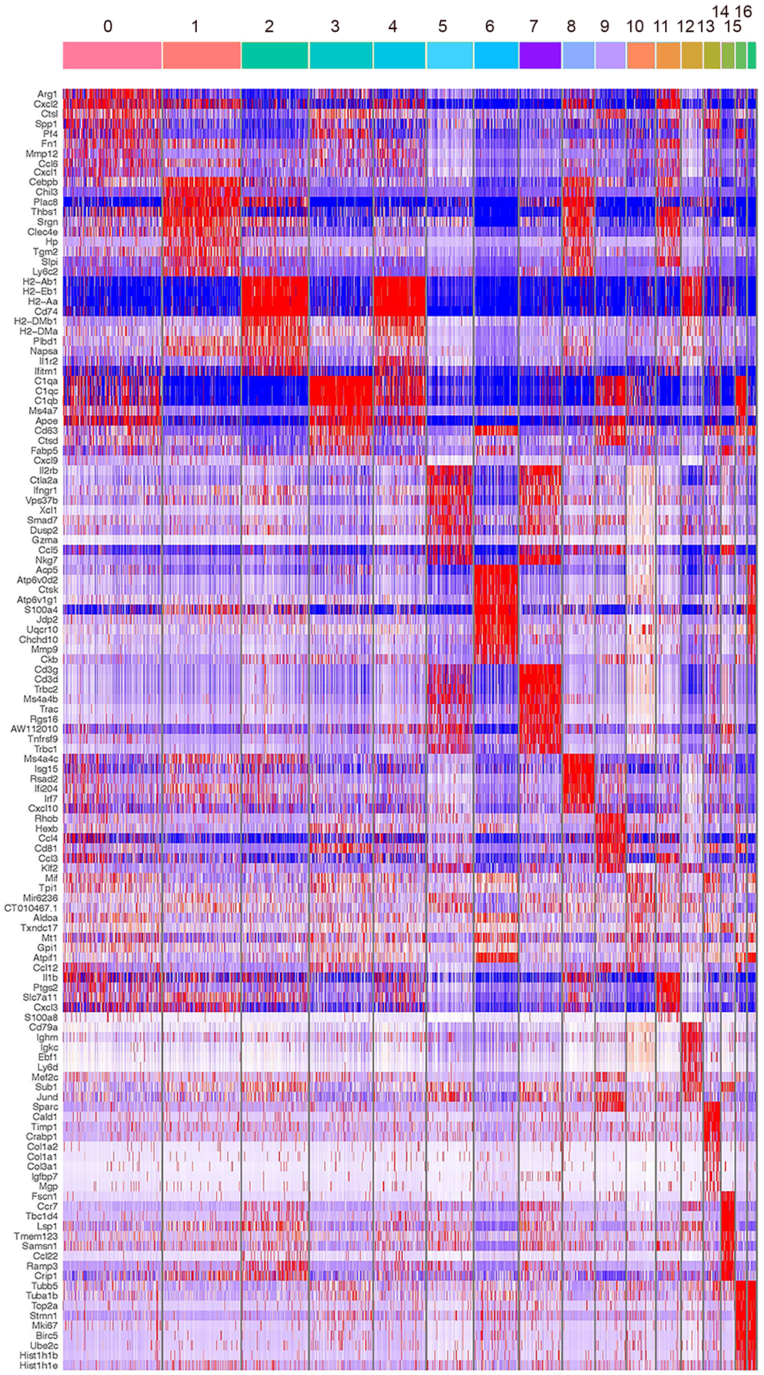

B

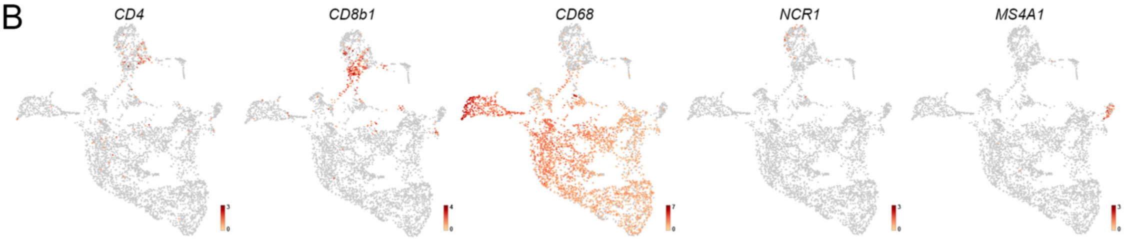

Supplementary Figure 10, related to Figure 5. scRNA-seq analysis reveals an altered immune landscape with myeloid specific PP2Ac deficiency in i.c. SB28 tumor. Heatmap displaying normalized expression of the top 10 up-regulated genes in each Seurat cluster. (B) Canonical markers were used to identified major CD45^{+} immune population.

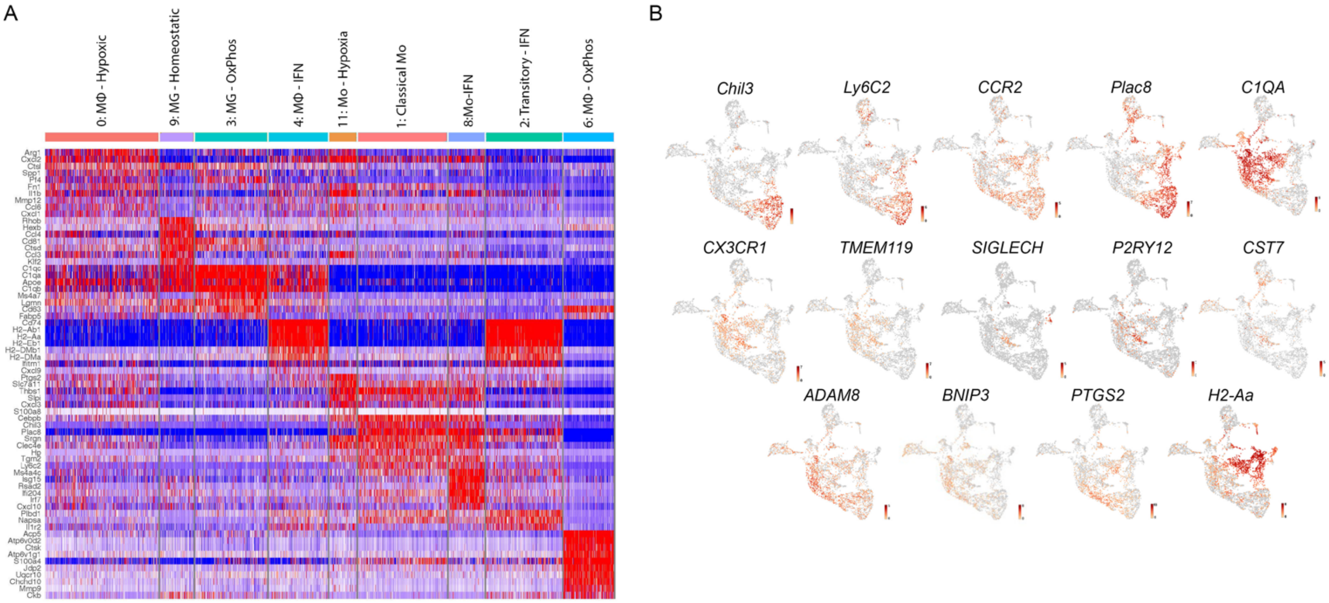

Supplementary Figure 11, related to Figure 5. scRNA-seq analysis of TAMS in i.c. SB28 tumor. (A) Heatmap displaying normalized expression of the top 10 up-regulated genes in each TAMs cluster. (B) UMAP showing key signature genes differentiating TAMs subsets.

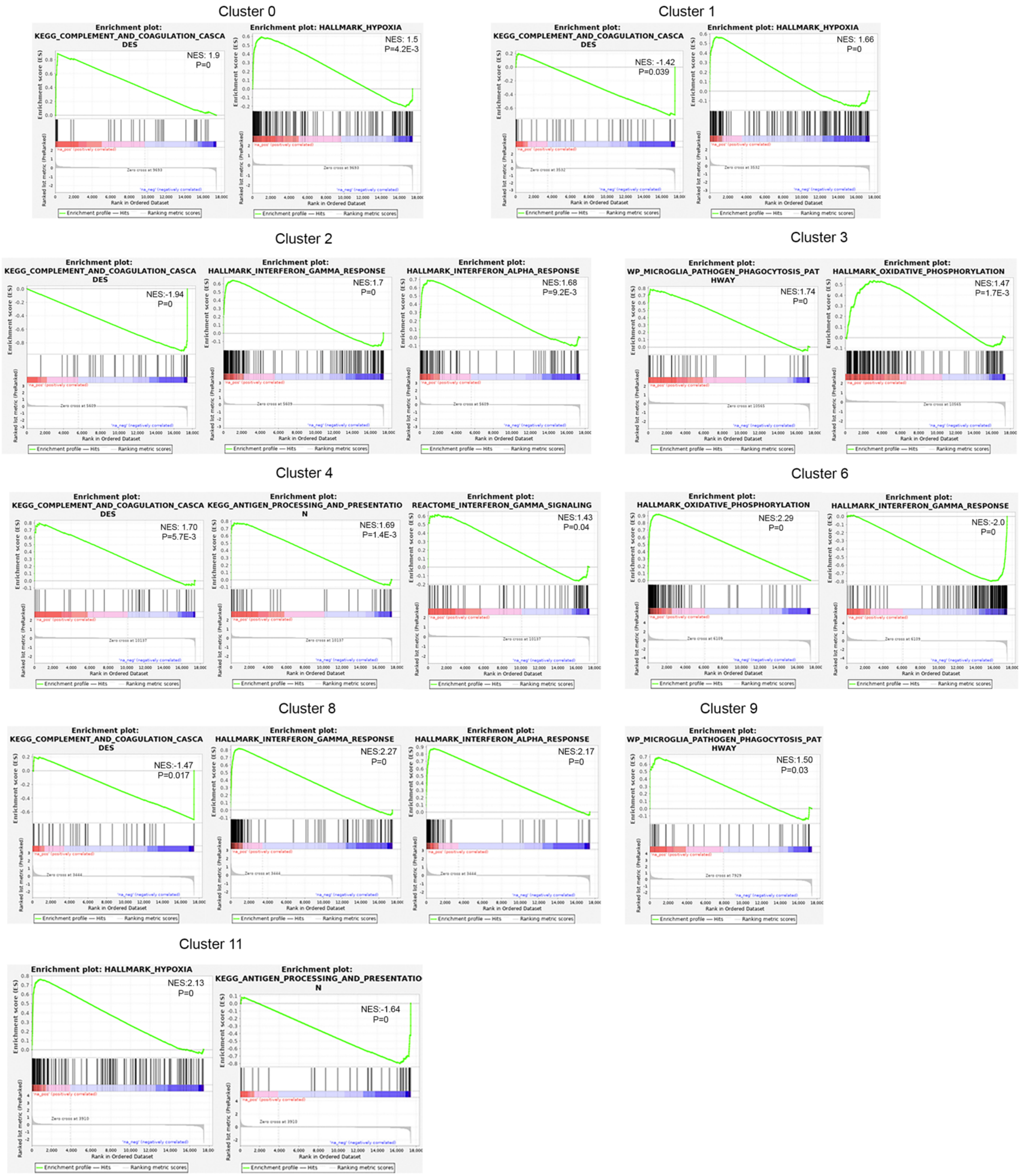

Supplementary Figure 12, related to Figure 5. Selected GSEA plots of significant gene signature pathway upregulated or downregulated in each TAMs cluster in scRNA-seq analysis of i.c. SB28 tumor.

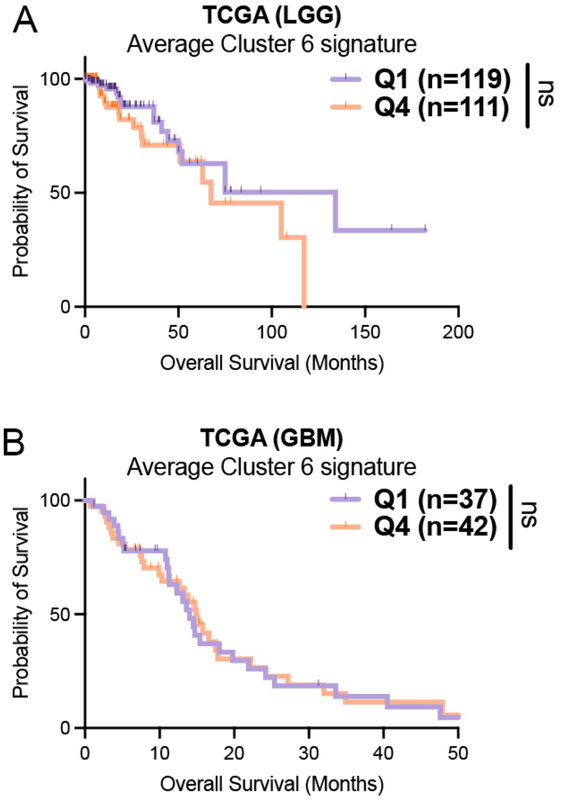

Supplementary Figure 13, related to Figure 5. Expression level of cluster 6 gene signature in patients does not independently predict survival in (A) GBM or (B) LGG patients. Mantel-Cox log-rank tests were used for survival analysis. (ns = non-significant)

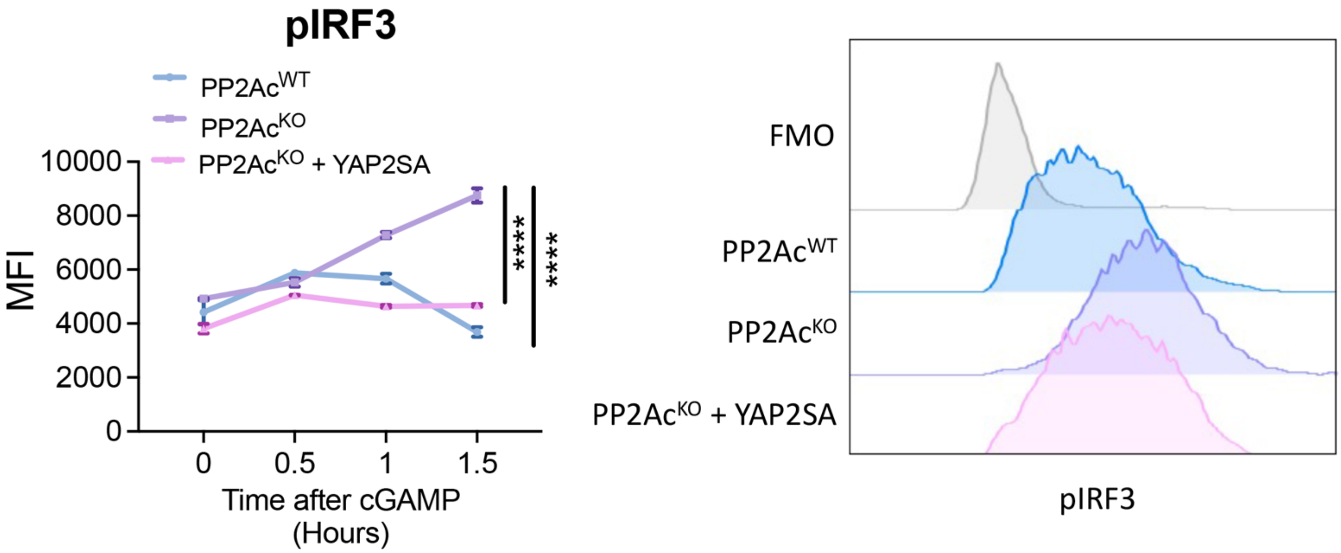

Supplementary Figure 14, related to Figure 7. PP2A^{KO}-mediated enhancement of pIRF3 activation is abolished by YAP overexpression. PP2Ac^{WT}, PP2Ac^{KO} and PP2Ac^{KO} with YAP overexpressed (YAP2SA) THP-1 cells derived macrophages were treated with cGAMP (10ug/ml). Phosphorylation of IRF3 was analyzed by flow cytometry at different time points after stimulation. Representative FACs plot at 1.5 hours after cGAMP treatment. Data are from one experiment. Error bars depict SEM. P values were calculated by one-way ANOVA with Tukey's multiple comparison test (****P<0.0001).

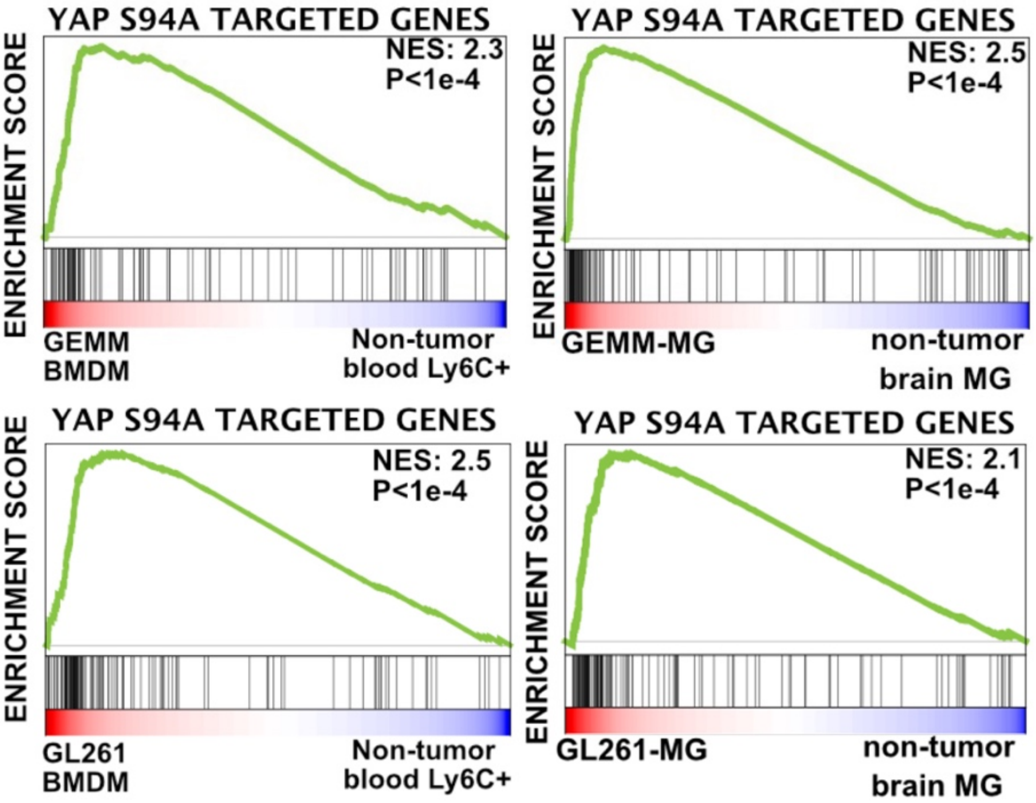

Supplementary Figure 15, related to Figure 7. YAPS94A gene signature is enriched in murine glioma associated Bone Marrow Derived Macrophage (BMDM) and microglia (MG) relative to normal tissue control. RNAseq dataset (Bowman et al., 2016) of sorted BMDM and MG from murine GBM (GL261 or Genetic Engineered Mouse Model (GEMM) we well as blood Ly6C$^+$ monocytes and nontumor MG were obtained. GSEA plots for YAPS94A targeted signature among GEMM BMDM vs blood monocytes, GEMM MG vs non tumor MG, GL261 BMDM vs blood monocytes, and GL261 MG vs non tumor MG.

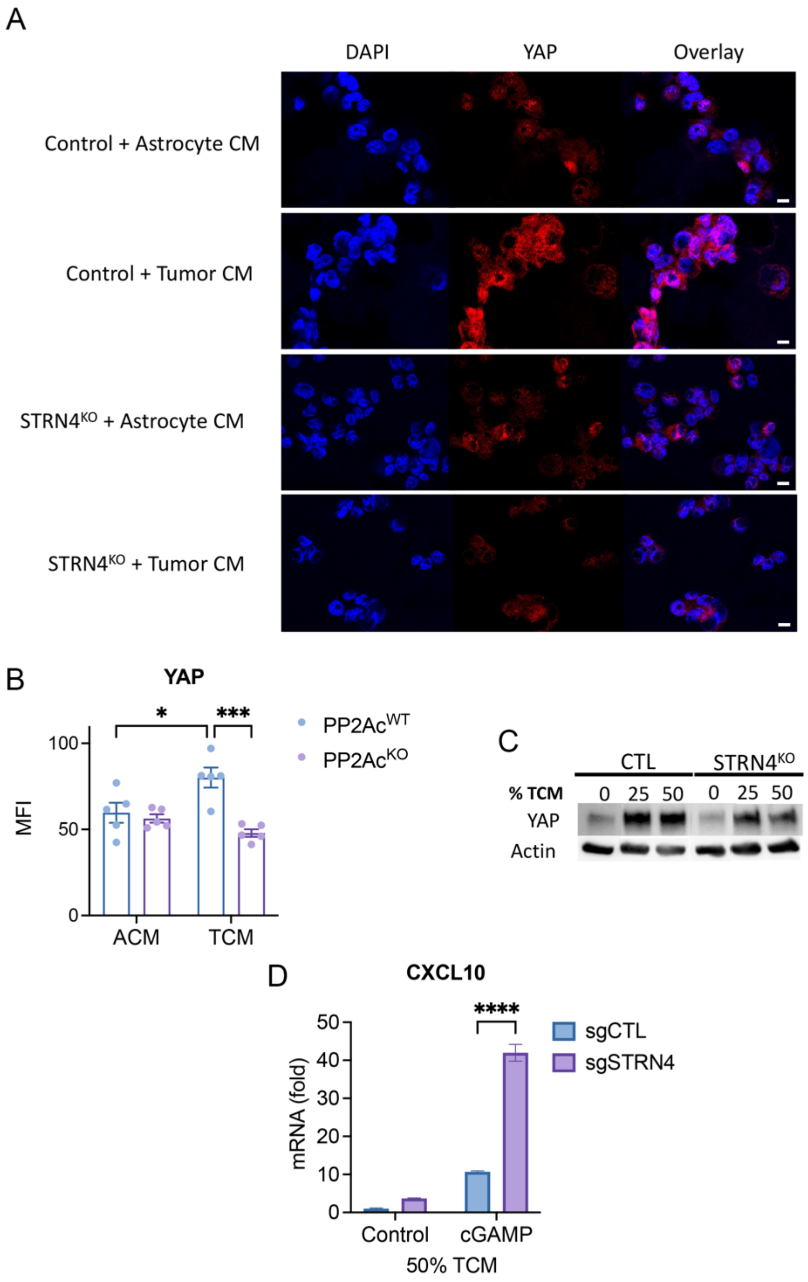

Supplementary Figure 16, related to Figure 7. GBM conditioned medium enhances YAP expression in THP-1 derived macrophages, which is partially reversed by STRN4^{KO}. CTL or STRN4^{KO} THP-1 cells were differentiated into macrophage under PMA for 24 hours. Cells were then rested for 24 hours before treated with 50% astrocytes-conditioned medium (ACM) or 50% GBM tumor-conditioned medium (TCM) conditioned medium for 5 days. (A) Histological analysis of STRN4^{WT} or STRN4^{KO} cells stained for YAP (red) and nucleus (4,6-diamidino-2-phenylindole (DAPI), blue). Scale bar, 10 µm. Each image is representative of n=4 independent images. (B) Quantification of YAP mean fluorescent intensity (MFI) within manually selected intracellular regions of interests. Data summary of n=4 independent images. (C) CTL or STRN4^{KO} derived macrophages were treated with increasing % of TCM. Protein expression was analyzed by immunoblotting. (D) After 5 days of TCM treatment, CTL or STRN4^{KO} derived macrophages were treated with cGAMP (10ug/ml) for 4 hours, expression of CXCL10 was measured via RT-qPCR. Error bars depict SEM. P values were calculated by one-way ANOVA with Tukey's multiple comparison test (*P<0.05, ***P<0.001, ****P<0.0001).
